# Comparative Efficacy of Platelet-Rich Plasma, Corticosteroid, Hyaluronic Acid, and Placebo (Saline) Injections in Patients with Lateral Elbow Tendinopathy: A Randomized Controlled Trial

**DOI:** 10.3390/jcm14020472

**Published:** 2025-01-13

**Authors:** Maciej Dejnek, Aleksandra Królikowska, Mateusz Kowal, Paweł Reichert

**Affiliations:** 1 Department of Orthopedics, Traumatology and Hand Surgery, Faculty of Medicine, Wroclaw Medical University, 50-556 Wroclaw, Poland; pawel.reichert@umw.edu.pl; 2 Physiotherapy Research Laboratory, University Centre of Physiotherapy and Rehabilitation, Faculty of Physiotherapy, Wroclaw Medical University, 50-556 Wroclaw, Poland; aleksandra.krolikowska@umw.edu.pl (A.K.); mateusz.kowal@umw.edu.pl (M.K.)

**Keywords:** tennis elbow, elbow tendinopathy, platelet-rich plasma, hyaluronic acid, glucocorticoids, injections, pain measurement

## Abstract

**Background**: Lateral elbow tendinopathy is a common condition that significantly alters the function of the upper extremities. In case of first-line treatment failure, different local injections are usually used. Due to the lack of sufficient evidence to support it, we conducted the study to compare the outcomes of different single injections, including Platelet-Rich Plasma (PRP), Corticosteroids (CS), Hyaluronic Acid (HA), and placebo (saline). **Methods:** Sixty patients with confirmed lateral elbow tendinopathy were enrolled in the study and divided into four groups. Pain intensity (average for the current day, at rest, during provocative tests) measured by Visual Analogue Scale (VAS), pressure pain threshold (PPT), Patient-Rated Tennis Elbow Evaluation (PRTEE), Disability of Arm, Shoulder and Hand (DASH), Subjected Elbow Value (SEV), and strength of selected muscle groups were measured before and during follow-up visits (1, 4, 12, 24, and 52 weeks after treatment). The treatment was considered successful when minimal clinically important difference (MCID) was achieved in primary outcomes (VAS, PRTEE). **Results**: A significant improvement was achieved in most measurements in all groups. At the final follow-up, MCID for the mean pain reduction measured with VAS and improvement in the PRTEE score were achieved in 52 and 54 patients, respectively. The complete absence of pain was achieved after 1, 4, 12, 24, and 52 weeks in 2, 5, 10, 22, and 40 patients, respectively. The comparison between the groups found a significant difference in pain intensity between CS and other groups one week after injection, between the CS and PRP group in the fourth week, and between PRP and HA in the fourth week (*p* < 0.05). No other significant differences were found between each group at each follow-up time point. **Conclusions**: We conclude that each injection treatment provides good long-term clinical outcomes, but not better than placebo. A CS injection might be regarded as a more effective treatment only within the first month post-injection.

## 1. Introduction

Lateral elbow tendinopathy is a common condition that can significantly affect upper limb function [1]. Commonly known as tennis elbow, it affects about 1–3% of the population, most between 35 and 50 years old [2,3]. The disease occurs in those who overload the limb by repeatedly performing wrist extension, forearm torsional movements, and forceful gripping [4]. In microscopic evaluation, angiofibroblastic tendinosis was described as the result of improper repetitive microtrauma healing [2].

In case of failure of the rehabilitation protocol, there is no evidence-based second-line therapy [5]. In everyday clinical practice, it is common to perform one of the injection procedures [6]. The biological mechanisms of these procedures are unclear and may even be contrary. These include, but are not limited to, injections of corticosteroids (CS), autologous platelet-rich plasma (PRP), and hyaluronic acid (HA). Despite their frequent use, there is much controversy about their clinical effectiveness, and more evidence-based data are required [5,7,8,9,10,11].

Autologous PRP injection is among the protocols most frequently studied for lateral elbow tendinopathy [12]. The idea behind this treatment is to locally administer a high volume of platelet-derived growth factors and cytokines responsible for tissue healing [13]. Many previous publications showed good outcomes and safety profiles of PRP injection [12]. However, there are many concerns related to the wide variety of PRP preparation and injection protocols that can lead to the administration of substances with entirely different molecular properties [12,14,15]. An additional concern is that, despite the good results of PRP injection in patients with lateral elbow tendinopathy, a similar efficacy can be obtained with saline injection alone [12,16,17].

CS injection is a well-known and frequently used treatment option leading to suppression of local inflammatory processes. Although lateral elbow tendinopathy is a degenerative condition with an inflammation phase only at the beginning, the use of CS provides quick pain relief for most patients. Studies have shown that CS injections are beneficial only for a short time, which usually disappears three months after treatment [18]. CS injection, with a higher rate of complication than other treatment options, was even proven to have a worse long-term effect than physical therapy or a wait-and-see policy [19,20].

HA is a high-molecular-weight, unbranched polysaccharide located throughout the body, especially in synovial fluid. Its safety has been widely proven in the treatment of different orthopedic conditions. Its main role is to maintain the structural and functional viscoelastic characteristics of tissues [21]. According to our knowledge, there are limited number of studies evaluating the efficacy of HA injection in tennis elbow, although most of them reported promising results [21,22,23].

Despite the frequent clinical use of these therapies, their comparative efficacy remains unclear. Current literature often focuses on comparing individual treatments to either an active comparator or placebo, leaving a gap in studies that directly compare multiple injection therapies, including PRP, CS, HA, and saline placebo, in the same patient population. Furthermore, studies have raised concerns that the benefits of PRP might be comparable to placebo, underscoring the need for more robust, head-to-head comparisons [12].

This study aimed to compare the clinical efficacy of three different injection therapies—PRP, CS, and HA—against placebo (saline injection) in patients with lateral elbow tendinopathy. It evaluated their effectiveness in reducing pain and improving functional outcomes over short-term and long-term follow-up periods.

## 2. Materials and Methods

### 2.1. Ethical Considerations

The study was carried out according to the Declaration of Helsinki and has the approval of the Bioethics Committee of Wroclaw Medical University (KB-26/2019, 21 January 2019). All patients agreed to participate in the study and signed an informed consent.

### 2.2. Study Design, Randomization and Allocation

The study was a single-center, double-blinded, prospective randomized control trial registered on clinicaltrials.gov under the ID: NCT04521387. The study was reported according to CONSORT guidelines [24]. We planned to enroll 120 patients with lateral elbow tendinopathy and divide them into four equal groups receiving an autologous platelet-rich plasma (PRP-Group; *n* = 30), corticosteroid (CS-Group; *n* = 30), or hyaluronic acid (HA-Group; *n* = 30) injection in the area of painful lateral epicondyle, or to the control, the placebo group (PL-Group; *n* = 30) receiving an injection of normal saline in the same area. Each patient received an identification number that was drawn from an opaque envelope. The list with random allocation of identification numbers to different groups had been previously prepared by the person who later administered the injection. In case of an unexpectedly insufficient number of patients willing to participate in the study due to the relatively short expiration date of the reagents for determining cytokines in PRP samples, a quasi-parallel way of patients allocation into groups was implemented. The envelopes were divided into two separate boxes. The first box had the first set of shuffled opaque envelopes with numbers randomly assigned to one of four groups with unequal proportions (PRP:CS:HA:PL = 30:10:10:10). After assigning envelopes from the first box, we planned to use the second one, which had the rest of the assignments in equal proportions (CS:HA:PL = 20:20:20).

In the first step, the patient drew an envelope with a number from the first box and then handed it to the person administering the injection, who reviewed its content and checked the number on a pre-prepared list for the given box to determine which injection corresponded to that number. Both the patient and the outcomes assessor did not know what kind of substance was used for the treatment. Only the person administering the injection had access to the patient identification number list and knew the exact type of treatment.

### 2.3. Deviations from Preregistered Protocol and Justifications

Unfortunately, the much lower-than-expected level of interest in participating in the study and the sudden introduction of considerable limitations in institutional functioning due to the SARS-CoV-2 pandemic regulations resulted in a significant extension of the recruitment time and ending the study prematurely (after the first box of opaque envelopes was completed), before a sufficient number of study participants were collected.

Another deviation from the preregistered protocol was number of questionnaires collected as secondary outcomes. Initially, we planned to collect many more questionnaires for functional evaluation (Mayo Elbow Performance Score, Oxford Elbow Score, and Quality of Life Questionnaire SF-36). However, the first few patients started complaining about the time needed to complete the questionnaires, so we stopped collecting them to not discourage patients from participating in the study.

### 2.4. Population

The inclusion criteria for the study were as follows: participants aged 20 to 60 years, persistent lateral elbow pain for a minimum duration of three months, confirmation of lateral epicondyle tendinopathy through at least one positive provocative test (Cozen’s, Thomson’s, Mill’s, Maudsley’s, or the chair test), no prior history of invasive treatments (including injections), and lack of improvement following rehabilitation. Exclusion criteria included neurological disorders affecting the upper limb, hematological conditions, diabetes, malignancies of the affected upper extremity, gout, advanced elbow osteoarthritis, previous surgeries involving the affected elbow, suspected infections, pregnancy, or the use of medications influencing platelet function or the coagulation system.

The patients were recruited from among those who volunteered to participate, which they learned about from the University’s website, social media, and during a visit to an outpatient clinic. The patients were assessed from February 2021 to June 2023 in the Department of Orthopedics, Traumatology and Hand Surgery at the Faculty of Medicine, Wroclaw Medical University.

Before the therapeutic intervention, patients had clinical and radiological examination and laboratory analysis of blood samples (complete blood count analysis). Elbow anterior-posterior and lateral view radiographs and ultrasound examination confirmed lateral elbow tendinopathy and excluded other pathologies. Patients were included in the study regardless of the severity of ultrasonographic changes characteristic for the lateral elbow tendinopathy (tendon edema, neovascularization, echogenicity disturbance, tendon tear, calcifications, or enthesis disruption).

### 2.5. Intervention

**Figure 1:**
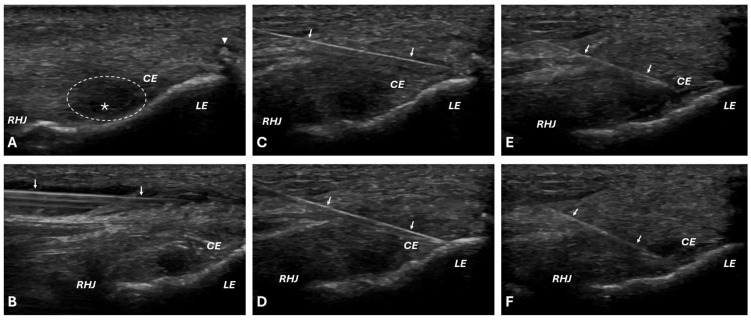
Presentation of the ultrasound-guided injection technique: (**A**) ultrasonographic image of pathological changes in lateral epicondyle tendinopathy (tendon edema, hypoechoic area, partial tear, bony spurs); (**B**) needle positioning during the administration of anesthetic; (**C**–**F**) injection using a multiple-needle puncture technique from proximal to distal, extending from the deep forearm fascia to the periosteum (**D**), with medication administered during needle withdrawal (**E**) and directly into the visibly damaged area (**F**). Arrow, needle position; arrowhead, bony spurs; asterisk, tendon tears; CE, common extensor origin; dotted-line circle, hypoechoic area; LE, lateral epicondyle; RCJ, radiohumeral joint.

Patients from the PRP-Group (n = 30) received a single injection of autologous platelet-rich plasma without activator (2 mL) in the area of attachment of the common extensor tendon under supervision of ultrasound and five minutes after the subcutaneous injection of local anesthetic (1 mL 1% lidocaine). PRP was prepared with the Mini GPS III Platelet Concentration System (Biomet Inc., Warsaw, IN, USA) according to the manufacturer’s protocol: 27 mL of blood drawn from each patient mixed with 3 mL of anticoagulant citrate dextrose solution A (ACD-A) and centrifugated for 15 min at 3200 revolutions per minute (RPM), with a force of 1740× *g*. Two mL of nonactivated liquid leukocyte-rich platelet-rich plasma (LR-PRP) was injected immediately after preparation (not longer than 20 min) into the common extensor (CE) origin using the peppering technique, proximal to distal from one access through the skin and with multiple punctures (approximately 10 times) through the deep forearm fascia to the periosteum with a 21G needle [25]. Part of the PRP sample (1 mL from 3 mL of prepared PRP) was taken for laboratory analysis of the levels of bioactive compounds (cell content, selected platelet-derived cytokines, and growth factors). A detailed description of the laboratory methodology was presented in a previous publication [26]. The applied ultrasound-guided injection technique is shown in Figure 1.

Patients from the CS-Group (n = 10) received a single injection of 2 mL of corticosteroid—betamethasone 7 mg/mL (Organon Group, Jersey City, NJ, USA). The patients in the HA-Group (n = 10) received a single injection of 2 mL 2% hyaluronic acid (40 mg) with mannitol (10 mg) from OST Tendon (TRB Chemedica, Carouge GE, Geneva, Switzerland). Saline (0.9% NaCl) injection was chosen as a placebo comparator for the PL-Group (n = 10) because of a similar impression for the patient compared to other injections. There are also some previously reported positive outcomes of that kind of intervention alone. All injections were administered by an experienced orthopedic specialist, and the injection technique for all groups was the same as described above for PRP-Group (aseptic conditions, local anesthetic, ultrasound guidance, peppering technique with a 21G needle in the area of proximal attachment of ECRB and CE).

Prior to the intervention, a medical history was collected for each patient, including information on comorbidities, medications, age, height, weight, symptom duration, hand dominance, type of work (physical labor), sports activity, and smoking status. Body Mass Index (BMI) was calculated based on height and weight. Sport activity was considered regular if it consisted of three or more training sessions per week.

All groups were instructed on how to perform daily stretching and strengthening exercises in the same manner. The training was unsupervised, and all patients were instructed on how to perform it. During the first week after injection, avoidance of overloading activities was recommended. Starting from the second week, daily stretching exercises for 15 min were recommended, including passive forced wrist flexion with the extended elbow (at least 10 rounds of 1 min each, with a 30 s interval). Two weeks after the intervention, patients were encouraged to start strengthening exercises of the forearm extensors, but only if the pain did not extend beyond 3 on the VAS during this activity. The simple technique for training eccentric extensors for the forearm was explained to all patients. During the exercises, the patient was advised to rest the pronated forearm on a table. Holding a bucket with weights using an overhand grip, the patient was instructed to passively dorsiflex the wrist and then slowly lower it actively. The recommended protocol was 10 rounds of 15 repetitions, with a 1 min rest between rounds. The starting weight was set by filling the bucket with 0.5 L of water (approximately 0.5 kg), with gradual increases every few days, ensuring that the exercises did not provoke pain greater than 3 on the VAS scale. The exercises were recommended to be performed for a duration of 2 months. The exercise instruction card for patients, translated into English, can be found in the Supplementary Data (Figure S1).

### 2.6. Primary Outcomes

The patients were evaluated before and 4, 12, 24, and 52 weeks after the intervention. After one week, we called patients by phone to ask about the average intensity of pain. The primary outcomes were the change in the pain intensity measured subjectively with the VAS scale and objectively with the algometer and the change in the Patient-Rated Tennis Elbow Evaluation questionnaire (PRTEE) during all follow-up intervals.

The VAS (Visual Analog Scale) is a graphical representation of a numerical scale ranging from 0 (no pain) to 10 (the worst pain imaginable). We recorded the average pain intensity on the current day, both at rest and during provocative tests. Five of the most commonly used provocative tests for tennis elbow were employed: Cozen’s test, Mill’s test, Maudsley’s lateral epicondylitis test, Thomson’s test, and the chair test [27,28,29]. The description of each test is presented in Table 1. Each test was considered positive if it caused an increase in the intensity of pain on the lateral side of the elbow joint.

The pressure pain threshold (PPT) was measured using the Wagner FPIX 25 Pain Test Algometer (Wagner Instruments, Riverside, CT, USA), a digital device with a 1 cm² hard rubber tip. The algometer was applied to the most tender spot in the epicondyle area and pressed at a rate of 1 newton (N) per second. The reading was taken when the patient reported that the sensation changed from touch to the slightest pain. A lower reading indicates greater tenderness, as less pressure is needed to cause pain. The procedure was repeated, and the final score was the average of two consecutive measurements for each elbow. The same rater conducted all the PPT assessments.

Among the patient-reported outcome measures (PROMs) designed for the functional assessment of patients with lateral elbow tendinopathy, we chose the validated and translated Polish version of the PRTEE questionnaire [30,31]. The PRTEE questionnaire is divided into two sections. The first section evaluates the intensity of pain with five questions, each rated on a scale from 0 (no pain) to 10 (worst imaginable pain). The second section assesses the function of the limb through 10 tasks, each rated from 0 (no difficulty) to 10 (unable to perform). The total score (ranging from 0 to 100) is calculated by adding the pain scale score to half of the functional scale score. A higher score indicates a worse function [30].

### 2.7. Secondary Outcomes

As the secondary outcomes, we assessed the Disability of Arm, Shoulder, and Hand Questionnaire (DASH), Subjected Elbow Value (SEV), and change in the strength of selected muscle groups.

The DASH questionnaire is one of the most common PROMs used in upper limb research. It includes 30 questions related to daily functioning. Patients respond to each question on a 5-point scale, with 1 representing the best result and 5 representing the worst. The total score is then converted to a 100-point scale, considering any unanswered questions by the patient. A higher score indicates a worse function of the upper extremity [32,33].

Patients were also asked to assess both elbows using the SEV, a validated, responsive, and easily administered tool for evaluating elbow condition [34]. The SEV is defined as the patient’s subjective assessment of the functionality of the elbow as a percentage, 100% representing a normal and healthy elbow.

We evaluated the change in strength of selected muscle groups assessed by hand-held dynamometers. The strength of elbow extensors and flexors, forearm supinators and pronators, and wrist extensors and flexors was performed using a microFET2 (Hoggan Scientific, Salt Lake City, UT, USA) dynamometer following the manufacturer’s recommendations. Grip and key-pinch strength assessments were performed using the BIMS Digital Grip (Baseline, Washington, DC, USA) and the pinch dynamometer (Baseline, Washington, DC, USA). Measurements were taken twice with a 1 min interval between each. Grip strength was measured with the patient seated comfortably, the elbow flexed at 90 degrees, and the forearm in a neutral position resting on the armrest. The patient was instructed to grip the dynamometer with maximum effort [35]. Similarly, the pressure force on a pinch dynamometer placed between the thumb and the bent fingers of the tested hand was assessed. If pain symptoms worsened, the patients were told to stop the test, and this was recorded.

Treatment effectiveness was defined as achieving a minimal clinically important difference (MCID) in pain reduction or an improvement in the PRTEE questionnaire scores between the baseline and follow-up periods. According to the literature, the MCID can be defined as 1.5 points for the VAS and 11 points for the PRTEE [36]. The number and types of adverse events and serious adverse events (defined as those that are fatal, life-threatening, or require hospitalization) were collected for each group.

### 2.8. Statistical Analysis

After data collection, statistical analyses were conducted, including both within-group and between-group comparisons. The within-group analysis included comparisons between pre-injection values and the respective follow-up time points. The Shapiro–Wilk test was conducted to evaluate the normality of the distribution of the results obtained. The significance of differences between groups at various follow-up stages for normally distributed values was tested using ANOVA (in the case of homogeneous variances) or Welch’s ANOVA (when variance homogeneity was not met), with post hoc testing performed using Tukey’s HSD or Games–Howell, respectively. For nonparametric data, the Kruskal–Wallis test was used, followed by Dunn’s post hoc test. Additionally, the analysis included Bonferroni correction for multiple comparisons (*p* < 0.0083). The significance of differences between pre-injection values and the respective follow-up time points within each group was tested using the Wilcoxon signed-rank test for nonparametric data and the paired *t*-test for parametric data. Bonferroni correction for multiple comparisons was applied in this analysis (*p* < 0.0125).

To achieve a significance level of 0.05, a power of 0.8, and to detect the MCID of 1.5 on the VAS scale with an assumed standard deviation of 2.0, each of the four groups was calculated to require approximately 24 participants. Accounting for an anticipated 20% patient drop-out rate, an additional 6 patients were added per group, resulting in a final total sample size of 120 patients.

Statistical analyses were performed with Statistica 13.3 software (TIBCO Software Inc, Pittsburgh, PA, USA), with significance set at *p* < 0.05 or adjusted according to the Bonferroni correction.

## 3. Results

### 3.1. Participants

After meeting the inclusion and exclusion criteria of 76 patients eligible for the study, we enrolled 60 patients aged 31 to 60 (48.03 ± 7.37) years who suffered lateral elbow pain for at least 3 months without improvement after rehabilitation. The characteristics of the patients are presented in Table 2. The One-Way ANOVA test or the Kruskal–Wallis test did not reveal significant differences between the groups in terms of age (*p* = 0.49), weight (*p* = 0.83), height (*p* = 0.97), Body Mass Index (BMI) (*p* = 0.74), duration of symptoms (*p* = 0.89), baseline DASH score (*p* = 0.20), baseline PRTEE score (*p* = 0.16), pain intensity at rest measured by VAS (*p* = 0.47), or grip (*p* = 0.87) or key-pinch strength (*p* = 0.60). There was a small, significant difference (*p* = 0.03) in the average pain intensity for the current day measured by VAS between the PRP-Group (5.07 ± 1.76) and CS-Group (6.75 ± 1.14) calculated in the post hoc Dunn test, which turned out to be non-significant after applying the Bonferroni correction (*p* < 0.0083).

**Figure 2:**
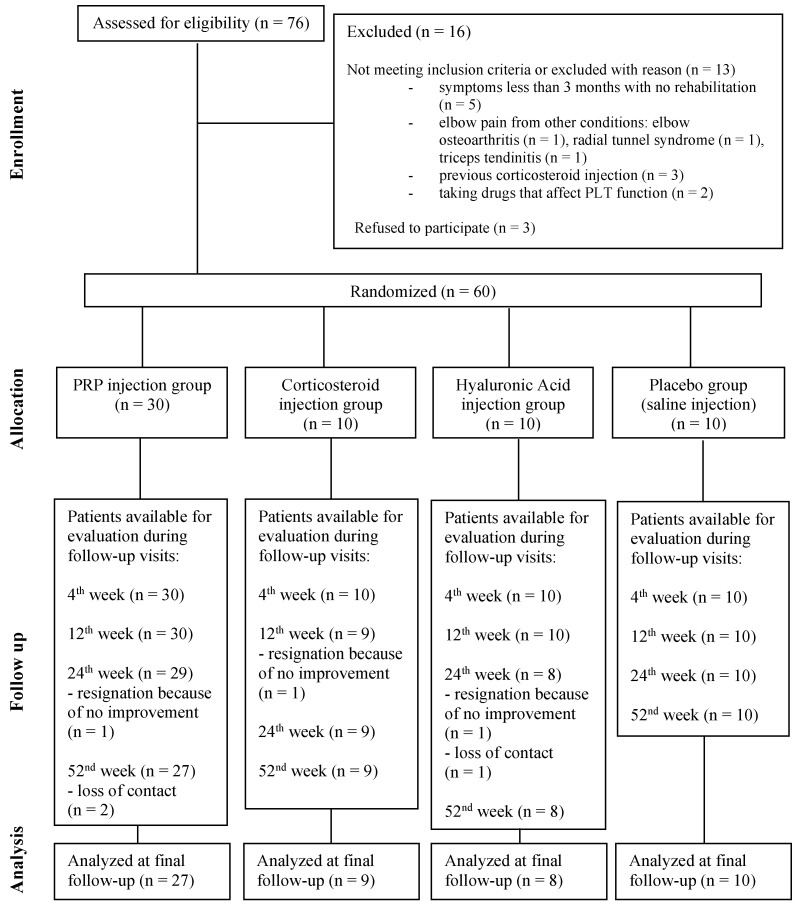
The CONSORT flow diagram showing the number of participants during the study.

During follow-up, all patients were available for evaluation 4 weeks after treatment. One patient did not return for follow-up after 12 weeks, four patients after 24 weeks, and six after one year. The main known reason for the absence of follow-up was a lack of improvement and change in treatment method (one patient from the PRP-Group and one from the CS-Group were referred to surgery, and a patient from the HA-Group received a corticosteroid injection). There was a loss of contact with three patients during the follow-up period, so we were unable to find the reason for withdrawal. At the final end point of the study, 54 patients were available for outcome evaluation, as shown on the flow chart in Figure 2.

### 3.2. Primary Outcomes

For all groups combined, there was a significant decrease in pain intensity measured with average VAS for the current day, VAS at rest, and VAS for all provocative tests between baseline and all follow-up points. After one week MCID for the average reduction in pain measured with VAS was achieved in 25 patients, but only in two cases the pain completely disappeared (both in the CS-Group). After 4, 12, 24, and 52 weeks, VAS MCID was achieved in 35, 43, 50, and 52 patients, respectively. The complete absence of pain was achieved after 4, 12, 24, and 52 weeks in 5, 10, 22, and 40 patients, respectively. A successful improvement in PROM, as indicated by changes in PRTEE scores, was observed in 30, 40, 53, and 54 patients at 4, 12, 24, and 52 weeks post-injection, respectively. Detailed results of the analysis divided into groups are presented in Table 3.

The statistical analysis using the Wilcoxon signed-rank test or the paired *t*-test showed significant reduction in pain measured with VAS (average pain for the current day, pain at rest, pain during provocative tests) between pre-treatment levels and each follow-up point in all groups combined. The tests revealed statistically significant differences in the VAS score between baseline (5.64 ± 1.76) and the 4th (3.41 ± 2.02), 12th (2.81 ± 2.19), 24th (1.54 ± 1.91), and 52nd (0.6 ± 1.37) weeks after injection in all groups together (*p* < 0.01). A similar improvement in VAS pain score decrease was observed in each group except in the following cases:

- In the PRP-Group when comparing baseline pain (5.07 ± 1.76) with pain after 1 week (4.25 ± 1.92) after applying the Bonferroni correction (*p* = 0.034);
- In the CS-Group when comparing baseline pain (6.75 ± 1.14) with pain after 12 (3.89 ± 2.15) and 24 weeks (2.28 ± 2.59) after applying the Bonferroni correction (*p* = 0.015, *p* = 0.011, respectively);
- In the HA-Group when comparing baseline pain (6.2 ± 1.75) with pain after 1 (4.90 ± 2.6), 4 (4.50 ± 1.72) and 52 weeks (0.0) after applying the Bonferroni correction (*p* = 0.038, *p* = 0.019, *p* = 0.012, respectively);
- In the PL-Group when comparing baseline pain (5.7 ± 1.81) with pain after 1 week (4.90 ± 2.23) after applying the Bonferroni correction (*p* = 0.22).

**Figure 3:**
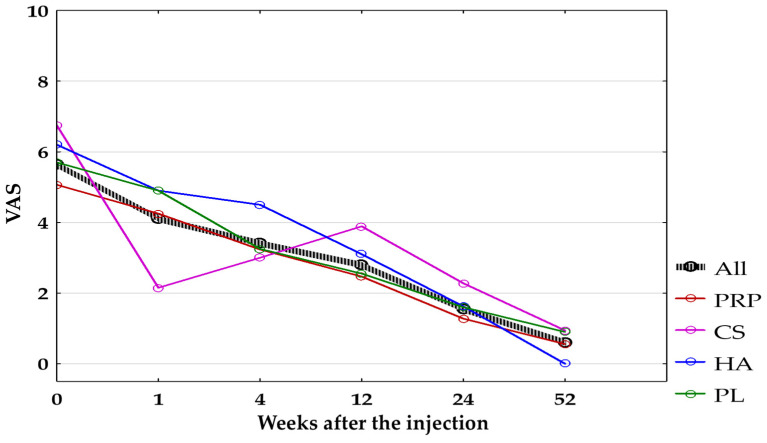
The average pain intensity for the current day measured with VAS for all groups combined and separated. All, all groups combined; CS, corticosteroid injection group; HA, hyaluronic acid injection group; PL, placebo injection group; PRP, platelet-rich plasma injection group; VAS, Visual Analogue Scale.

The comparison between the groups using the Kruskal–Wallis signed-rank test with post hoc Dunn test showed significant differences in pain reduction measured with VAS (average for the current day) only at the 1st week between CS-Group (2.15 ± 2.0) and other groups—PRP, HA, and PL (4.25 ± 1.92, 4.9 ± 2.6, 4.9 ± 2.23, respectively, with *p* < 0.05)—which turned out to be non-significant after applying the Bonferroni correction (*p* < 0.0083). The graphic representation of the average VAS change in all groups (combined and separated) is presented in Figure 3.

The decrease in pain at rest measured with VAS was also significant between baseline (3.51 ± 2.49) and all follow-up points in all groups combined (*p* < 0.0125). Non-significant differences were found after applying the Bonferroni correction (*p* < 0.0125):

- In the CS-Group when comparing baseline pain (4.5 ± 1.58) with pain after 24 weeks (1.11 ± 2.09; *p* = 0.018);
- In the HA-Group when comparing baseline pain (3.85 ± 1.8) with pain after 4 weeks (2.80 ± 2.04; *p* = 0.03);
- In the PL-Group when comparing baseline pain (3.0 ± 2.98) with pain after 4 (1.90 ± 2.73), 12 (1.70 ± 2.63), 24 (1.0 ± 1.94), and 52 weeks (0.5 ± 1.08) (*p* = 0.11, *p* = 0.04, *p* = 0.03, *p* = 0.03, respectively).

**Figure 4:**
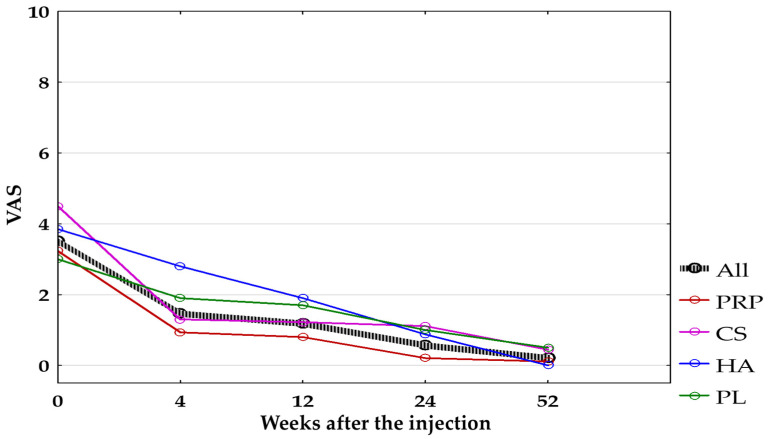
Pain intensity at rest measured with VAS for all groups combined and separated. All, all groups combined; CS, corticosteroid injection group; HA, hyaluronic acid injection group; PL, placebo injection group; PRP, platelet-rich plasma injection group; VAS, Visual Analogue Scale.

The VAS pain score at rest differs significantly only between the PRP-Group and HA-Group 4 weeks after treatment (0.93 ± 1.66 vs. 2.8 ± 2.04, *p* < 0.05), which turned out to be non-significant after applying the Bonferroni correction (*p* < 0.0083). The graphic representation of the average VAS change for the groups (combined and separated) is presented in Figure 4.

During provocative tests for all groups, combined pain decreased significantly (*p* < 0.0125) at all follow-up points. A statistically non-significant (after the Bonferroni correction *p* < 0.0125) improvement in pain during provocative tests was observed in the following groups:

- In the PRP-Group when comparing baseline pain during chair test with pain after 4 weeks (*p* = 0.014);
- In the CS-Group when comparing baseline pain during Mill’s test after 12, 24, and 52 weeks (*p* = 0.02);
- In the HA-Group when comparing baseline pain with pain after 4, 12, 24, and 52 weeks during Cozen’s test (*p* = 0.03, *p* = 0.013, *p* = 0.018, *p* = 0.018) and Mill’s test (*p* = 0.44, *p* = 0.06, *p* = 0.04, *p* = 0.02); when comparing baseline pain with pain after 4 weeks during Thomson’s test (*p* =0.16) and Maudsley’s test (*p* = 0.08); when comparing baseline pain with pain after 4 and 12 weeks during the chair test (*p* = 0.21, *p* = 0.02)
- In the PL-Group when comparing baseline pain with pain after 4 weeks during Thomson’s test (*p* = 0.025); when comparing baseline pain with pain after 4 and 12 weeks during Maudsley’s test (*p* = 0.02, *p* = 0.08) and the chair test (*p* = 0.04, *p* = 0.02).

**Figure 5:**
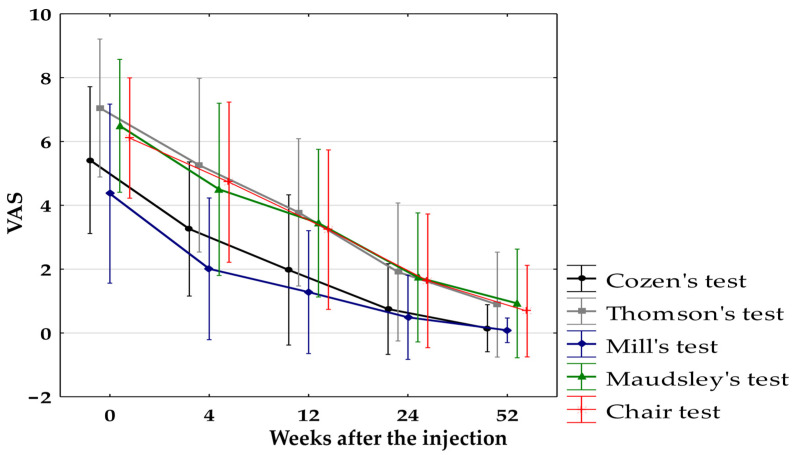
Pain intensity during provocative tests measured with VAS for all groups combined. VAS, Visual Analogue Scale.

The VAS pain score during Thomson’s provocative test was significantly different between the PRP-Group and CS-Group in the 4th week (6 ± 2.51 vs. 3.15 ± 2.54; *p* < 0.05), which turned out to be non-significant after applying the Bonferroni correction (*p* < 0.0083). No significant differences were found between the groups in other provocative tests or at other follow-up time points. The VAS changes during provocative tests during follow-up for all groups combined are presented in Figure 5.

Algometric evaluation of PTT at the site of the lateral epicondyle showed significant improvement (after applying Bonferroni correction *p* < 0.0125) when comparing baseline with PTT after 12, 24, and 52 weeks in all groups combined and after 24 and 52 weeks in the PRP-Group. Non-significant improvement was found in the following cases:

- In all groups combined: baseline vs. 4 weeks (*p* = 0.045);
- In the PRP-Group: baseline vs. 4 weeks (*p* = 0.10) and 12 weeks (*p* = 0.03);
- In the CS-Group: baseline vs. 4, 12, 24, and 52 weeks after treatment (*p* = 0.06, *p* = 0.82, *p* = 0.015, *p* = 0.03, respectively);
- In the HA-Group: baseline vs. 4, 12, 24, and 52 weeks after treatment (*p* = 0.68, *p* = 0.82, *p* = 0.02, *p* = 0.03, respectively);
- In the PL-Group: baseline vs. 4, 12, 24, and 52 weeks after treatment (*p* = 0.96, *p* = 0.29, *p* = 0.07, *p* = 0.02, respectively).

**Figure 6:**
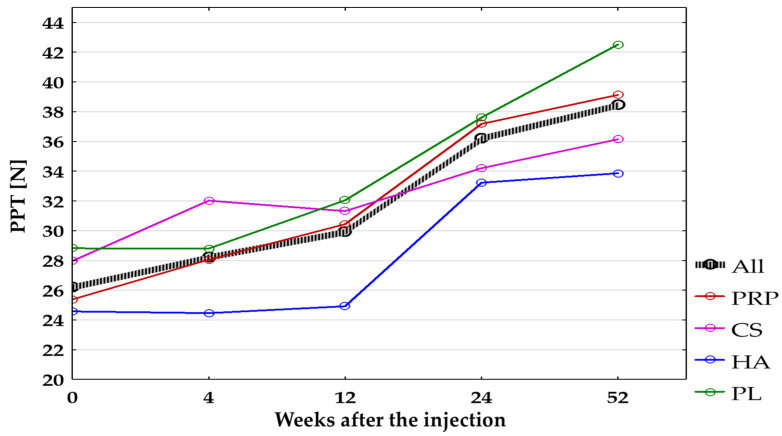
The Pressure Pain Threshold (N) at the site of the affected lateral epicondyle for all groups combined and separated. All, all groups combined; CS, corticosteroid injection group; HA, hyaluronic acid injection group; PL, placebo injection group; PPT, Pressure Pain Threshold; PRP, platelet-rich plasma injection group.

No significant differences between groups were found during all follow-up visits (Figure 6).

**Figure 7:**
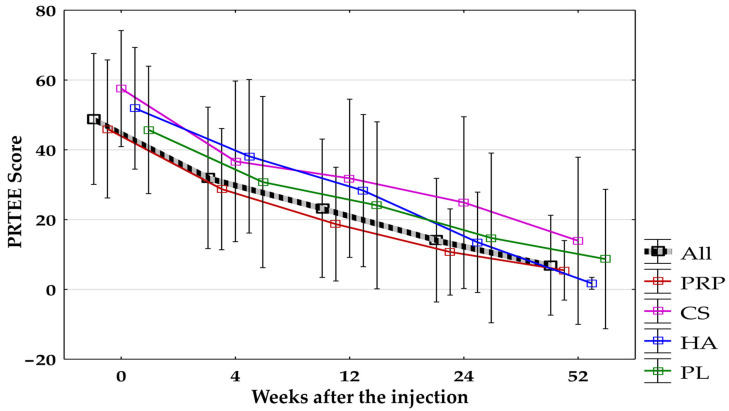
The PRTEE score for all groups combined and separated. All, all groups combined; CS, corticosteroid injection group; HA, hyaluronic acid injection group; PL, placebo injection group; PRP, platelet-rich plasma injection group; PRTEE, Patient-Rated Tennis Elbow Evaluation.

The PRTEE score decreased significantly (*p* < 0.0125) during all follow-up time points compared to baseline in all groups combined and separated. No significant differences were found between groups during all follow-up visits (Figure 7).

A detailed description of basic characteristics and all primary outcomes for all groups with number of participants, mean and standard deviation, median, Q1–Q2, and minimal and maximal values are presented in Supplementary Data (Table S1).

### 3.3. Secondary Outcomes

The grip strength of the affected limb improved significantly (*p* < 0.0125) in all groups combined between baseline and 12, 24, and 52 weeks after treatment. Improvement in grip strength was also observed in the following cases:

- In the PRP-Group between baseline and 24 and 52 weeks after treatment (*p* < 0.0125), and between baseline and 12 weeks with *p* = 0.034 (non-significant after the Bonferroni correction);
- In the CS-Group between baseline and 4 weeks after treatment with *p* = 0.03, which turned out to be non-significant after applying the Bonferroni correction;
- In the HA-Group between baseline and 24 and 52 weeks after treatment (*p* < 0.0125), and between baseline and 12 weeks after treatment with *p* = 0.018, which turned out to be non-significant after applying the Bonferroni correction;
- In the PL-Group between baseline and 24 and 52 weeks after treatment (*p* < 0.0125), and between baseline and 12 weeks after treatment with *p* = 0.015, which turned out to be non-significant after applying the Bonferroni correction.

**Figure 8:**
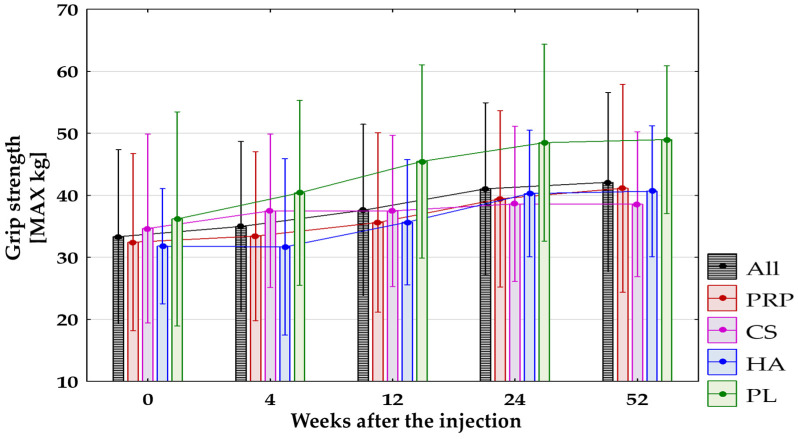
The grip strength (MAX kg) for all groups combined and separated. All, all groups combined; CS, corticosteroid injection group; HA, hyaluronic acid injection group; PL, placebo injection group; PRP, platelet-rich plasma injection group.

No significant differences between groups were found during all follow-up visits. Figure 8 presents changes in grip strength in all groups.

**Figure 9:**
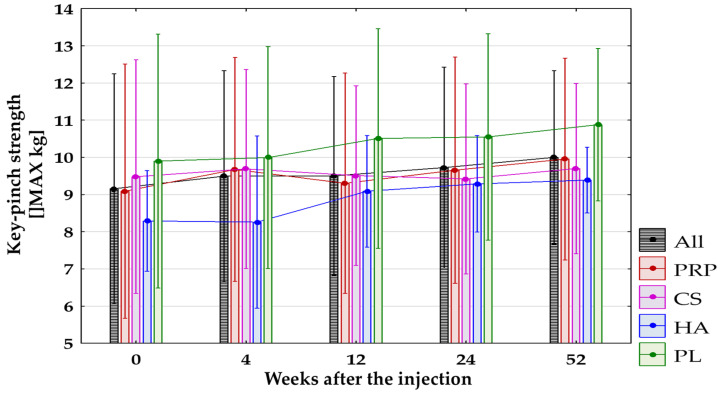
The key-pinch strength (MAX kg) for all groups combined and separated. All, all groups combined; CS, corticosteroid injection group; HA, hyaluronic acid injection group; PL, placebo injection group; PRP, platelet-rich plasma injection group.

The key-pinch strength significantly improved in all groups combined between the baseline and 12 (*p* = 0.02, non-significant after the Bonferroni correction), 24, and 52 weeks after treatment (*p* < 0.0125). No significant differences in change of key-pinch strength were found between the baseline and follow-up time points in CS and PL groups. Some significant improvement in key-pinch strength was observed in the PRP-Group between the baseline and 4 (*p* = 0.02, non-significant after the Bonferroni correction) and 52 weeks after treatment (*p* < 0.0125). Also, in the HA-Group, a difference between baseline and 52 weeks was found with *p* = 0.03, which turned out to be non-significant after the Bonferroni correction. No significant differences between groups were found during all follow-up visits. Changes in key-pinch strength in all groups are presented in Figure 9.

During the evaluation of the change in the strength of selected muscle groups using a hand-held dynamometer, we found an improvement in elbow flexion for all groups combined and for the PRP-Group 52 weeks after treatment (*p* < 0.0125). When the strength of elbow extension was evaluated, no changes were found during follow-up in all groups. The strength of wrist extension improved significantly in all groups combined at 12, 24, and 52 weeks after treatment (*p* < 0.001). It also improved in the PRP-Group after 12 (*p* < 0.0125), 24, and 52 weeks (*p* < 0.001), in the CS-Group after 4 and 52 weeks (*p* < 0.0125), and in the HA-Group at 24 and 52 weeks after treatment (*p* < 0.0125). The strength of wrist flexion improved significantly in all groups combined at 24 and 52 weeks after treatment (*p* < 0.001), in the PRP-Group after 52 weeks (*p* < 0.0125), in the CS-Group after 12 weeks (*p* < 0.0125), and in the HA-Group after 24 and 52 weeks (*p* < 0.0125). No significant improvement in wrist flexion was observed in the PL-Group at all follow-up points. The strength of forearm supination improved significantly after 12, 24, and 52 weeks in all groups together (*p* < 0.001). It also improved in the PRP-Group and PL-Group after 24 and 52 weeks (*p* < 0.0125) and in the CS-Group at 52 weeks after treatment (*p* < 0.0125). Although there was a significant improvement in the strength of the forearm pronation at 12, 24, and 52 weeks after treatment when all groups were combined and in the PRP-Group (*p* < 0.001), there was no improvement at all in the CS, HA, and PL groups during the study. No significant differences between groups were found during all follow-up visits. All results of the strength of selected muscle groups with statistical significance of the comparison between baseline and follow-up time points are presented in Table 4.

**Figure 10:**
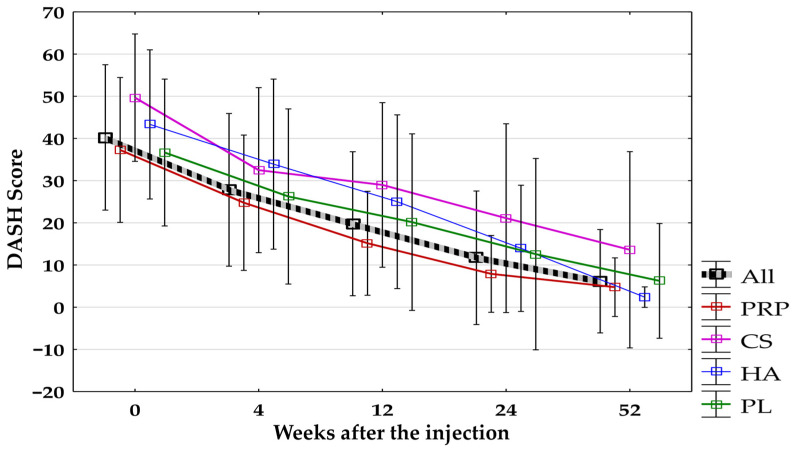
The DASH score for all groups combined and separated. All, all groups combined; CS, corticosteroid injection group; DASH, Disability of Arm, Shoulder, and Hand Questionnaire; HA, hyaluronic acid injection group; PL, placebo injection group; PRP, platelet-rich plasma injection group.

A significant decrease in the DASH score compared to baseline and each follow-up period was observed in the groups counted together and separately. Only in the HA-Group after applying the Bonferroni correction did the comparison between baseline and after 4 weeks turn out to be non-significant (*p* = 0.02). No significant differences between groups were found during all follow-up visits. The changes in the DASH score in all groups are presented in Figure 10.

**Figure 11:**
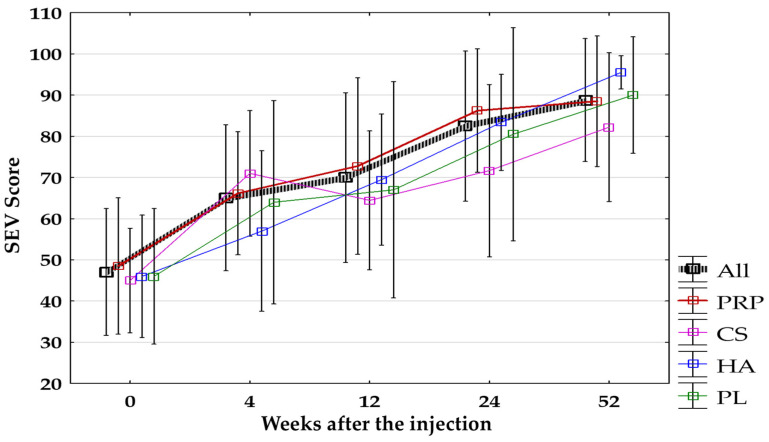
The SEV score (%) for all groups combined and separated. All, all groups combined; CS, corticosteroid injection group; HA, hyaluronic acid injection group; PL, placebo injection group; PRP, platelet-rich plasma injection group; SEV, Subjected Elbow Value.

A significant increase in the SEV score compared to baseline was observed at every follow-up point for all groups together (*p* < 0.0125) and separate. After applying the Bonferroni correction comparison between baseline and after 4 weeks in the HA-Group (*p* = 0.04), and between baseline and after 4 and 12 weeks in the PL-Group (*p* = 0.03, *p* = 0.02, respectively) turned out to be non-significant. No significant differences between groups were found during all follow-up visits. The increase in SEV is shown in Figure 11.

A detailed description of all results of secondary outcomes for all groups with number of participants, mean and standard deviation, median, Q1-Q2, and minimal and maximal values are presented in Supplementary Data (Table S2).

### 3.4. Complications

There were no serious adverse events in any of the groups throughout the study. No infections, neural lesions, collateral ligaments disruptions, tendon ruptures or joint cartilage damage were found during the whole follow-up period. The main side effect was the intensification of pain compared to baseline, which was observed in the following cases:

- In five patients (17%) after a week in the PRP-Group, in one patient (10%) in the CS-Group, in one (10%) in the HA-Group, and in three patients (30%) in the PL-Group.
- In four patients (13%) after a month in the PRP-Group, in one patient (10%) in the PL-Group, and in zero patients in groups CS and HA,
- In two patients (7%) after 12 weeks in the PRP-Group, in one patient (11%) in the CS-Group, and in zero patients in groups HA and PL,
- In zero patients after 24 weeks in groups PRP and HA, in two patients (22%) in the CS-Group, and in one patient (10%) in the PL-Group,
- In zero patients after 52 weeks in any group.

Among the patients experiencing pain intensification after 12 weeks, one (from the PRP-group) opted for surgical treatment, while another (also from the PRP-group) chose to change their employment to a role that did not involve lifting heavy objects.

## 4. Discussion

Although most of the patients had some ongoing improvement measured by MCID of VAS (>72%) and PRTEE (>81%) after 3 months and more in all groups, complete pain disappearance was a rare result. After one week, only two patients after CS injection had complete recovery, which worsened for one patient during the second follow-up visit. After 12 weeks, pain disappeared in only about 10% of patients from injection groups compared to placebo, which had the best result (40%). The logical explanation for that could be tissue irritation followed by local inflammation caused by injected substances, but there is no evidence of that in the literature. After 24 weeks, full recovery in injection groups increases equally to about 30% (saline injection—60%). After a year, we observed a lack of pain in about 74% of all available patients, with the worst result (about 50%) in the CS-Group.

More detailed analysis showed significant improvement in most measurements (VAS, VAS at rest, VAS during provocative tests, PTT, PRTEE score, DASH score, SEV score, grip strength, and strength of wrist flexion, extension, supination, and pronation). The pattern of improvement was similar to most of them: improved outcomes in 4 weeks after CS injection, which dropped significantly after 3 months and continued to grow similarly to other methods, including saline injection. Statistical analysis did not show significant differences between groups except those found after 1 week (higher VAS pain decrease in the CS-Group) and after 4 weeks (lower pain during the Thomson’s test measured with VAS in the CS-Group).

A recent meta-analysis based on 31 good-quality trials comparing one type of injection therapy (CS, PRP, autologous blood, and botulinum toxin) with a placebo in tennis elbow treatment shows similar findings [10]. Tavassoli et al. found greater pain relief and functional improvement after CS injection versus placebo within the first month after treatment but no significant differences after 3 and 6 months after injection. They also did not find significant improvement comparing placebo with PRP and autologous blood injection at any follow-up time. None of the treatments were significantly better than placebo in improving strength during all follow-up time points [10].

Different results were previously presented in another metanalysis by Dong et al. based on 27 randomized control trials presenting results 6 months after injection treatment [11]. The authors observed that HA injections led to a greater reduction in pain compared to other injections included in the study (CS, HA, PRP, autologous blood, botulinum toxin, glycosaminoglycan polysulfate, and prolotherapy), with the exception of prolotherapy. They also reported that the third most effective treatment was PRP injection with the peppering technique. In the Bayesian network meta-analysis, all studied types of injections were better than placebo (saline injection and wait-and-see policy), but only HA and prolotherapy had significant superiority. The study also concluded that CS injection is a suboptimal choice for lateral elbow tendinopathy, but short-term results (less than 6 months) were not investigated [11]. Those results were not confirmed by our study, in which all outcomes were comparable to placebo (saline injection) 6 months after treatment.

The autologous PRP injection is among the most commonly used treatment for tennis elbow [12]. Evaluation of its efficacy is quite challenging because of the wide variety of PRP preparation protocols resulting in different quality of the final product [14,37]. From the molecular point of view, it is expected to obtain different outcomes depending on the platelets density, the presence of leukocytes, the contamination of red blood cells, the fibrin content, the type of activation of platelets degranulation, and the injection technique used for treatment. An additional aspect of our study involved evaluating the correlations between blood components, growth factors, and cytokines with the clinical effectiveness of PRP injection treatment for lateral elbow tendinopathy. We demonstrated there that a higher platelet concentration in PRP correlates with a greater content of growth factors, which, in turn, correlates with more remarkable clinical improvement. Because of the complexity of this subject and to increase the transparency and readability of the collected data, we presented those results in detail in a separate paper [26].

For a more accurate evaluation of PRP treatment, a consensus has been developed on what information should be described in the studies [38]. The mean platelets concentration in the PRP in our study was more than 1 million per µL, which fulfills the most common definition of the PRP [39]. The mean amount of leukocytes in the PRP used in our study was 30 × 103/μL, which places it among leukocyte-rich PRP. Despite the ongoing discussion of the positive or negative role of white blood cells in PRP, the recent meta-analysis showed no significant differences in outcomes between leucocyte-rich and leucocyte-poor PRP in the management of lateral epicondylitis [40]. Red blood cell contamination in our PRP was, on average 1 × 10^6^/μL, but there are no studies proving that this influences clinical effectiveness. The good clinical outcomes of PRP injection were proven by the meta-analysis delivered by Niemiec et al. [36]. In their work based on 26 studies, ongoing improvement of different PROMs was reported from 4 to 52 weeks after injection. The study was focused on the achievement of MCID during follow-up points, and the findings were similar to those in our study. Unfortunately, no comparison to placebo injection or wait-and-see policy was performed, so it is not certain whether improvement was related to the injection itself or the natural history of the disease [36]. When PRP injection treatment was compared to placebo (saline injection, lidocaine injection, dry needling), most studies did not report significant improvement during follow-up [41,42,43,44,45].

As mentioned above, CS injection is efficient in improving the clinical outcomes of lateral elbow tendinopathy only in the short term, which has been proven in many studies [10,18,46]. Smidt et al., in their randomized controlled trial, suggest that despite the better short-term results of CS injection, there was a higher success rate in the physiotherapy or wait-and-see policy groups [19]. Also, a higher rate of recurrence was observed after CS injection compared to other methods [20]. These findings were confirmed by our study, in which CS injection gives significantly greater pain relief only during the first four weeks, with the largest difference after one week. We also observed the lowest complete recovery rate in this group after 52 weeks. Additional findings indicate that CS should be contraindicated for long-term cases, as demonstrated by lower rates of symptom improvement or resolution in patients treated with CS compared to those undergoing other non-surgical treatments [47].

Only a few studies examined the effectiveness of HA injection in lateral elbow tendinopathy [21,23,48,49]. All of those studies showed a beneficial clinical effect of that treatment in terms of pain relief and improvement in PROM and grip strength. The proposed mechanism of action includes the reduction of pro-inflammatory markers, the improvement of tenocyte viability, and tendon repair [48]. Petrella et al., in their study, compared two HA injections (with a one-week interval) in 331 patients versus two placebo (normal saline) injections in 166 patients. They found a significantly better improvement in the HA group during all follow-up time points (30, 90, and 356 days from injection), including VAS at rest, VAS after grip strength measurement, grip strength, patient global satisfaction, patient assessment of normal function, and physician’s global assessment of elbow. Although there was some improvement in the control group, it was much smaller and ended on the second follow-up visit [21]. We did not obtain similar results in our study, in which HA was not favorable compared to any other injection, including a placebo. The possible reason for that could be a different injection protocol (two injections vs. one) and a different population (only athletes vs. general population) in their study. Apaydin et al., in their study, compared the effects of HA injection versus prolotherapy and found a significant improvement in pain at rest, pain with activity, and the Q-DASH score during 12 weeks of follow-up [49]. The size of improvement of these variables was comparable to those found in our study. Compared to our study, a similar pattern of improvement in pain decrease, Q-DASH, and PRTEE was reported in the study performed by Zinger et al. In their study, 35 patients were divided into two groups. One group received three HA injections (with a one-week interval), and the second group received three placebo (normal saline) injections. Contrary to our study, they had a large loss of patients in the control group during follow-up, so a group comparison was not possible [48]. Despite the good initial results of HA injection in lateral elbow tendinopathy treatment, more studies of good quality are needed to prove its value and establish a proper treatment protocol.

Most studies in which normal saline injection was used as a placebo found some beneficial effect of its use in terms of clinical improvement in patients with tennis elbow [41,43,44]. Similar results were obtained compared to PRP or autologous blood injection and even better in the long term compared to CS injection [10,50,51,52]. This suggests that the proper injection technique could be more important than the injected substance itself. Bisset et al., in their RCT, compared physiotherapy, SC injection, and wait-and-see policy for tennis elbow. They found that CS injection has the best results only in the first six weeks, physiotherapy between six and fifty-two weeks, and after fifty-two weeks, no difference between physiotherapy and the wait-and-see policy was found [53]. Finally, Vukelic et al., in their review, stated that placebo injections improve lateral epicondylopathy at high rates and that other injections (PRP, botulinum toxin, autologous blood, HA, and prolotherapy) are not superior to it [54].

Increasingly, authors emphasize the role of mechanical intervention during injections as an effective treatment method. In a review recently presented by Ricci et al., the potential therapeutic mechanisms of action for three commonly performed ultrasound-guided injection techniques were discussed: peritendinous hydrodissection detaching the fascia from the underlying common extensor tendon, intratendinous fenestration of the degenerated area, and scraping of the tendon–bone junction [55]. Using the aforementioned terminology, it can be observed that in our study, all three techniques were employed simultaneously during the injections, regardless of the substance administered.

By injecting the anesthetic, the subcutaneous tissue was separated from the forearm fascia, partially disrupting the connections between neovessels and neonerves originating from the subcutaneous tissue and extending toward the tendon. Repeated fenestrations through the entire thickness of the tendon resulted in partial cutting of disorganized fibers and local bleeding, thereby stimulating inflammatory processes. Additionally, multiple punctures of the periosteum caused irritation of the enthesis, although deliberate detachment or drilling of the enthesis was not performed.

The core focus of our study was the comparison of different injection substances rather than injection techniques. The lack of significant differences in outcomes, combined with favorable clinical results, suggests that the mechanical aspect of the procedure may have played a primary role. One limitation of our study was the absence of a control group treated without invasive methods, which would have helped evaluate the impact of mechanical intervention alone on clinical outcomes.

The primary limitation of this study was the lower-than-anticipated participant enrollment, which impacted the planned sample size and group allocation. Initially, the study aimed to recruit 120 patients with lateral elbow tendinopathy, evenly distributed across four groups receiving PRP, CS, HA, or placebo (30 patients per group). However, limited patient interest and institutional restrictions due to the SARS-CoV-2 pandemic led to an extended recruitment period and a premature study conclusion. To adapt, a quasi-parallel allocation was implemented, using an initial set of opaque envelopes to assign patients to groups with an unequal distribution (PRP:CS:HA:PL = 30:10:10:10). Although a second envelope set with equal assignments was planned, enrollment concluded before this phase.

Despite these adjustments, deviations from the initial protocol were clearly documented, ensuring transparency regarding the modifications. This transparency helps to clarify the study’s methodological decisions and supports the credibility of the findings, despite the uneven group sizes and underpowered comparisons [56,57]. Future studies with a larger, multi-center design may provide stronger statistical power to detect potential differences between treatments.

To further understand the role of injection therapies in lateral elbow tendinopathy, high-quality studies are needed to evaluate both single and multiple injections, as well as to compare these interventions with non-invasive treatments such as physical therapy and exercise programs. Additionally, future research should focus on identifying patient characteristics that may predict a positive response to specific injection treatments, thereby improving patient selection and targeting those most likely to benefit. Establishing standardized protocols for treatment administration, outcome measurement, and follow-up duration will also help clarify the therapeutic potential of PRP, CS, and HA in managing this condition.

## 5. Conclusions

A single PRP, CS, HA, or placebo injection provided similar long-term outcomes (≥ 12 weeks) for patients with lateral elbow tendinopathy. While corticosteroid injections offered greater pain relief in the short term, particularly within the first month, this effect was not sustained. Studied injections demonstrated comparable effectiveness in pain reduction and functional improvement, indicating limited long-term benefit. However, results should be interpreted cautiously due to the study’s main limitation: a lower-than-planned number of participants led to unequal group distributions and underpowered comparisons. A larger sample size may help reveal more significant differences between treatment groups.

## Figures and Tables

**Table 1:** Description of provocative tests performed in the study.

Provocative Test	Initial Position	Pain-Provoking Activity
Cozen’s test	Elbow in 90 flexion, forearm fully pronated	Active wrist extension against resistance
Thomson’s test	Elbow in full extension, forearm fully pronated	Active wrist extension against resistance
Mill’s test	Elbow in 90 flexion, forearm fully pronated	Active wrist supination against resistance
Maudsley’s lateral epicondylitis test	Elbow in full extension, forearm fully pronated	Active third finger extension against resistance
Chair test	Elbow in full extension, forearm fully pronated, holding the backrest of the chair	Lifting the chair by holding its backrest

**Table 2:** Baseline characteristics of the patients involved in the study.

Study Group	All	PRP-Group	CS-Group	HA-Group	PL-Group
*n*	60	30	10	10	10
Age [years]	48.03 (7.37)	49.03 (6.12)	47.6 (8.81)	48.6 (6.9)	44.9 (9.76)
Female–male ratio					
[*n*]	29:31	15:15	5:5	6:4	3:7
BMI [kg/m^2^]	27.66 (4.64)	27.54 (4.67)	28.92 (5.05)	26.61 (4.66)	27.79 (4.53)
Symptoms duration					
[months]:					
Mean (SD):	16.57 (35.24)	17.33 (25.82)	10 (11.05)	6.9 (6.39)	30.5 (73.9)
Median (Q1–Q3):	5 (3–12)	5 (3–18)	5 (4–9)	5 (3–8)	5 (3–12)
MIN–MAX:	3–240	3–120	3–36	3–24	3–240
Elbow affected					
[*n* (%)]:					
Right:	39 (65%)	19 (63%)	6 (60%)	7 (70%)	7 (70%)
Left:	21 (35%)	11 (37%)	4 (40%)	3 (30%)	3 (30%)
Dominant hand affected					
[*n* (%)]	42 (70%)	22 (73%)	6 (60%)	7 (70%)	7 (70%)
Physical labor					
[*n* (%)]	29 (48%)	15 (50%)	4 (40%)	5 (50%)	5 (50%)
Regular sport activity					
[*n* (%)]	23 (38%)	14 (47%)	3 (30%)	3 (30%)	3 (30%)
Current smokers					
[*n* (%)]	7 (12%)	3 (10%)	1 (10%)	2 (20%)	1 (10%)

If not mentioned otherwise, the values are presented as arithmetic mean (standard deviation). BMI, Body Mass Index; CS, corticosteroids; HA, hyaluronic acid; *n*, number of patients; PL, placebo; PRP, platelet-rich plasma; Q, quartiles; SD, standard deviation.

**Table 3:** Number and ratio of patients with improvement in primary outcomes during the study.

	Follow-Up [Weeks]	PRP-Group	CS-Group	HA-Group	PL-Group
Number of patients with MCID in pain reduction measured with VAS [*n* (%)]	1	9 (30)	9 (90)	4 (40)	3 (30)
	4	15 (50)	8 (80)	4 (40)	8 (80)
	12	20 (67)	7 (78)	8 (80)	8 (80)
	24	25 (86)	8 (89)	8 (100)	9 (90)
	52	26 (96)	9 (100)	8 (100)	9 (90)
Number of patients with complete pain reduction: VAS = 0 [*n* (%)]	1	0	2 (20)	0	0
	4	2 (7)	1 (10)	0	2 (20)
	12	4 (13)	1 (11)	1 (10)	4 (40)
	24	10 (34)	3 (33)	3 (38)	6 (60)
	52	19 (70)	5 (56)	8 (100)	8 (80)
Number of patients with MCID in PRTEE score improvement [*n* (%)]	4	18 (60)	7 (70)	5 (50)	5 (50)
	12	25 (83)	8 (89)	7 (70)	8 (80)
	24	28 (97)	8 (89)	8 (100)	9 (90)
	52	27 (100)	9 (100)	8 (100)	10 (100)

CS, corticosteroid; HA, hyaluronic acid; MCID, minimal clinically important difference; *n*, number of patients; PL, placebo; PRP, platelet-rich plasma; PRTEE, Patient-rated Tennis Elbow Evaluation; VAS, Visual Analogue Scale; %, the percentage of patients among those examined during a given follow-up.

**Table 4:** The strength of muscle groups before treatment and during follow-up visits.

	Group	Baseline	4 Weeks	*p* ^1^	12 Weeks	*p* ^2^	24 Weeks	*p* ^3^	52 Weeks	*p* ^4^
Elbow flexion	All	242.95 (89.74)	237.90 (85.70)	0.27	246.39 (85.28)	0.64	260.38 (88.81)	**0.04**	268.43 (96.18)	≤0.001 *
	PRP	234.97 (90.34)	236.83 (91.14)	0.81	246.44 (84.57)	**0.** **03**	254.63 (93.82)	**0.03**	258.19 (92.92)	0.01 *
	CS	237.55 (71.30)	220.16 (62.00)	0.25	232.25 (73.57)	0.28	242.17 (75.17)	0.99	243.53 (88.01)	0.87
	HA	236.44 (69.63)	219.59 (78.65)	0.10	219.36 (73.27)	0.15	255.22 (65.97)	0.57	279.39 (82.99)	**0.** **02**
	PL	278.80 (121.83)	277.14 (94.44)	0.90	285.96 (104.38)	0.59	297.53 (102.03)	0.29	309.74 (119.79)	0.13
Elbow extension	All	176.00 (59.44)	172.90 (49.71)	0.51	177.43 (54.01)	0.55	182.40 (57.08)	0.37	181.99 (55.28)	0.16
	PRP	175.96 (66.71)	172.07 (54.51)	0.43	172.17 (56.83)	0.39	176.89 (57.05)	0.52	177.08 (57.04)	0.80
	CS	171.94 (28.81)	175.70 (34.55)	0.43	178.75 (39.13)	0.10	186.00 (47.01)	0.05	173.14 (35.13)	0.43
	HA	154.23 (40.87)	150.25 (35.53)	0.55	162.25 (44.46)	0.29	163.26 (34.43)	0.65	173.35 (33.79)	**0.02**
	PL	201.92 (70.91)	195.25 (55.11)	0.50	207.21 (60.96)	0.67	210.45 (74.74)	0.52	210.11 (74.28)	0.45
Wrist flexion	All	158.01 (50.26)	159.41 (43.64)	0.51	165.57 (51.15)	**0.** **014**	173.66 (49.43)	≤0.001 *	175.71 (49.34)	≤0.001 *
	PRP	159.03 (61.28)	151.81 (48.27)	0.30	162.07 (59.05)	0.40	174.03 (60.40)	**0.02**	175.26 (60.08)	**0.** **01** *
	CS	143.26 (19.99)	168.84 (39.88)	**0.** **03**	152.06 (21.10)	**0.** **01** *	159.10 (27.45	**0.02**	159.54 (36.43)	0.06
	HA	153.56 (34.69)	160.29 (33.87)	0.24	164.59 (39.43)	0.13	174.93 (23.96)	0.01 *	172.44 (17.50)	0.01 *
	PL	174.17 (47.92)	171.91 (42.12)	0.86	189.20 (53.24)	0.14	184.66 (46.00)	0.48	194.11 (42.89)	0.07
Wrist extension	All	114.05 (49.92)	126.82 (45.44)	**0.02**	140.71 (47.19)	≤0.001 *	157.32 (47.26)	≤0.001 *	169.08 (48.64)	≤0.001 *
	PRP	116.44 (52.47)	121.50 (51.14)	0.80	140.46 (49.45)	**0.** **01** *	159.56 (51.16)	≤0.001 *	172.99 (53.26)	≤0.001 *
	CS	110.36 (19.54)	136.90 (25.59)	**0.** **01** *	122.47 (29.51)	0.11	133.28 (28.64)	**0.02**	147.89 (30.18)	**0.** **01** *
	HA	97.66 (34.63)	113.90 (52.74)	0.19	133.37 (43.39)	**0.** **02**	158.05 (47.7)	**0.** **01** *	160.62 (44.65)	0.01 *
	PL	127.00 (73.24)	145.63 (29.76)	0.44	165.22 (46.88)	0.08	171.87 (47.00)	0.05	184.37 (50.58)	**0.** **01**
Forearm supination	All	24.48 (10.75)	25.40 (10.64)	0.55	28.63 (13.25)	≤0.001 *	31.51 (11.43)	≤0.001 *	32.18 (11.86)	≤0.001 *
	PRP	26.05 (12.13)	24.71 (10.54)	0.31	29.05 (15.74)	0.08	32.05 (14.39)	≤0.001 *	31.19 (15.11)	**0.** **01** *
	CS	25.48 (6.96)	31.33 (10.87)	0.17	26.68 (7.30)	0.46	31.62 (5.94)	**0.02**	33.48 (4.19)	**0.** **01** *
	HA	20.81 (7.11)	20.23 (11.08)	0.77	24.60 (4.92)	**0.** **04**	26.15 (5.85)	**0.** **04**	28.48 (7.15)	**0.** **03**
	PL	22.46 (12.45)	26.67 (8.42)	0.20	33.17 (14.66)	0.05	34.13 (7.84)	**0.** **004** *	36.64 (8.62)	0.01 *
Pronation of the forearm	All	41.36 (18.71)	43.70 (17.83)	**0.04**	46.40 (17.18)	≤0.001 *	50.02 (20.40)	≤0.001 *	51.37 (18.14)	≤0.001 *
	PRP	36.64 (18.35)	40.24 (17.93)	0.09	46.44 (20.15)	≤0.001 *	48.44 (22.56)	≤0.001 *	49.46 (19.84)	≤0.001 *
	CS	47.69 (16.26)	49.98 (16.64)	0.21	46.64 (12.41)	0.64	51.32 (15.45)	0.19	50.21 (14.62)	0.37
	HA	41.81 (15.39)	41.29 (16.82)	0.83	43.75 (13.06)	0.58	45.23 (12.73)	0.82	49.53 (11.65)	0.13
	PL	48.74 (22.98)	50.21 (18.74)	0.68	48.68 (16.52)	0.99	57.27 (23.06)	0.09	59.03 (20.61)	0.10

Values are presented as arithmetic mean (standard deviation) in N. *p*-value represents the significance of the comparison between time periods: *p* ^1^ = baseline vs. 4th week; *p* ^2^ = baseline vs. 12th week; *p* ^3^ = baseline vs. 24th week; *p* ^4^ = baseline vs. 52nd week (*p* < 0.05 are highlighted in bold; *p* < 0.0125, considered significant according to the Bonferroni correction, are marked with an asterisk). All, all groups combined; CS, corticosteroid injection group; HA, hyaluronic acid injection group; PL, placebo injection group; PRP, platelet-rich plasma injection group.

## Data Availability

The data used to support the findings of this study are available in the Supplementary Files. Additional data are available from the corresponding author upon reasonable request.

## Supplementary Materials

The following supporting information can be downloaded at: https://www.mdpi.com/article/10.3390/jcm14020472/s1, Figure S1: the exercise instruction card for patients; Table S1: primary outcomes; Table S2: secondary outcomes.
