# Supplementary material for: Comparative Efficacy of Platelet-Rich Plasma, Corticosteroid, Hyaluronic Acid, and Placebo (Saline) Injections in Patients with Lateral Elbow Tendinopathy: A Randomized Controlled Trial
Source: J Clin Med. 2025 Jan 13;14(2):472. doi: 10.3390/jcm14020472 (PMC11766060; doi:10.3390/jcm14020472)
Supplement: Supplementary file 1 [file jcm-14-00472-s001.zip › jcm-3339471-supplementary.pdf]

Supplementary materials - Table S1: primary outcomes

	Follow-up (weeks)	unit	Group	*n*	Mean	SD	Median	Minimum	Maximum	Q1	Q2
age	0	year	All	60	48.03	7.37	49.50	31.00	60.00	44.00	53.50
body weight	0	kg	All	60	82.55	17.92	80.00	50.00	130.00	69.00	93.50
body height	0	m	All	60	1.72	0.09	1.72	1.50	1.94	1.67	1.79
BMI	0	kg/m^2	All	60	27.66	4.64	27.05	18.94	38.40	24.11	30.38
duration of symptoms	0	month	All	60	16.57	35.24	5.00	3.00	240.00	3.00	12.00
VAS	0		All	60	5.64	1.76	6.00	2.00	9.00	4.75	7.00
VAS	1		All	60	4.12	2.26	4.00	0.00	10.00	2.00	5.75
VAS	4		All	60	3.41	2.02	3.00	0.00	9.00	2.00	5.00
VAS	12		All	59	2.81	2.19	2.00	0.00	10.00	1.50	4.00
VAS	24		All	56	1.54	1.91	1.00	0.00	9.00	0.00	2.25
VAS	52		All	54	0.60	1.37	0.00	0.00	7.00	0.00	1.00
VAS at rest	0		All	60	3.51	2.49	4.00	0.00	9.00	2.00	5.00
VAS at rest	4		All	60	1.47	1.96	0.00	0.00	8.00	0.00	2.50
VAS at rest	12		All	59	1.20	1.85	0.00	0.00	7.00	0.00	2.00
VAS at rest	24		All	56	0.59	1.37	0.00	0.00	6.00	0.00	0.00
VAS at rest	52		All	54	0.22	0.74	0.00	0.00	3.00	0.00	0.00
PPT	0	N	All	60	26.25	12.03	23.19	3.63	67.96	19.56	30.44
PPT	4	N	All	60	28.24	11.98	25.74	9.61	72.57	20.30	31.06
PPT	12	N	All	59	29.91	12.70	27.65	10.89	72.47	21.57	34.13
PPT	24	N	All	56	36.21	11.95	34.32	12.75	71.29	28.64	40.80
PPT	52	N	All	54	38.48	11.62	36.87	14.34	68.65	30.40	46.09
Cozen's test	0	VAS	All	60	5.42	2.30	5.00	0.00	10.00	5.00	7.50
Cozen's test	4	VAS	All	60	3.26	2.10	2.75	0.00	7.50	2.50	5.00
Cozen's test	12	VAS	All	59	1.97	2.35	1.00	0.00	10.00	0.00	3.00
Cozen's test	24	VAS	All	56	0.75	1.42	0.00	0.00	5.00	0.00	1.00
Cozen's test	52	VAS	All	54	0.15	0.74	0.00	0.00	5.00	0.00	0.00
Thomson's test	0	VAS	All	60	7.05	2.16	7.50	1.00	10.00	6.00	8.00
Thomson's test	4	VAS	All	60	5.26	2.72	5.75	0.00	10.00	2.50	7.50
Thomson's test	12	VAS	All	59	3.78	2.31	4.00	0.00	8.00	2.50	6.00
Thomson's test	24	VAS	All	56	1.91	2.16	1.00	0.00	7.50	0.00	3.00
Thomson's test	52	VAS	All	54	0.89	1.64	0.00	0.00	8.00	0.00	1.00
Mill's test	0	VAS	All	60	4.37	2.81	5.00	0.00	10.00	2.50	7.50
Mill's test	4	VAS	All	60	2.01	2.22	2.00	0.00	7.50	0.00	3.00
Mill's test	12	VAS	All	59	1.28	1.93	0.00	0.00	8.00	0.00	2.00
Mill's test	24	VAS	All	56	0.49	1.32	0.00	0.00	7.00	0.00	0.00
Mill's test	52	VAS	All	54	0.08	0.39	0.00	0.00	2.50	0.00	0.00
Maudsley's test	0	VAS	All	60	6.49	2.08	7.00	1.00	10.00	5.00	7.50
Maudsley's test	4	VAS	All	60	4.50	2.70	5.00	0.00	10.00	2.50	7.50
Maudsley's test	12	VAS	All	59	3.44	2.31	3.00	0.00	8.00	2.00	5.00
Maudsley's test	24	VAS	All	56	1.74	2.02	1.00	0.00	7.50	0.00	3.00
Maudsley's test	52	VAS	All	54	0.93	1.70	0.00	0.00	8.00	0.00	1.00

Chair test	0	VAS	All	60	6.11	1.88	7.00	0.00	10.00	5.00	7.50
Chair test	4	VAS	All	60	4.73	2.51	5.00	0.00	10.00	2.50	7.00
Chair test	12	VAS	All	59	3.24	2.50	3.00	0.00	8.00	1.00	5.00
Chair test	24	VAS	All	56	1.63	2.09	1.00	0.00	7.50	0.00	2.75
Chair test	52	VAS	All	54	0.69	1.44	0.00	0.00	6.00	0.00	1.00
PRTEE	0		All	60	48.85	18.76	47.50	14.50	93.50	35.25	60.00
PRTEE	4		All	60	31.97	20.26	27.25	1.00	84.00	16.75	47.25
PRTEE	12		All	59	23.25	19.82	17.00	0.50	84.00	9.50	31.00
PRTEE	24		All	56	14.12	17.72	6.75	0.00	81.00	2.00	21.25
PRTEE	52		All	54	6.94	14.30	1.50	0.00	74.50	0.00	5.00
age	0	year	PRP	30	49.03	6.12	50.00	35.00	60.00	46.00	54.00
body weight	0	kg	PRP	30	82.40	19.80	79.50	50.00	130.00	68.00	95.00
body height	0	m	PRP	30	1.72	0.11	1.73	1.50	1.94	1.64	1.78
BMI	0	kg/m^2	PRP	30	27.54	4.67	26.90	18.94	38.40	23.74	30.42
duration of symptoms	0	month	PRP	30	17.33	25.82	5.00	3.00	120.00	3.00	18.00
VAS	0		PRP	30	5.07	1.76	5.00	2.00	9.00	4.00	6.00
VAS	1		PRP	30	4.25	1.92	4.00	2.00	10.00	3.00	5.00
VAS	4		PRP	30	3.23	1.89	3.00	0.00	8.00	2.00	4.00
VAS	12		PRP	30	2.47	2.08	2.00	0.00	10.00	1.00	3.00
VAS	24		PRP	29	1.28	1.37	1.00	0.00	5.00	0.00	2.00
VAS	52		PRP	27	0.56	1.05	0.00	0.00	4.00	0.00	1.00
VAS at rest	0		PRP	30	3.23	2.74	3.00	0.00	9.00	1.00	5.00
VAS at rest	4		PRP	30	0.93	1.66	0.00	0.00	5.00	0.00	1.00
VAS at rest	12		PRP	30	0.80	1.49	0.00	0.00	5.00	0.00	1.00
VAS at rest	24		PRP	29	0.21	0.68	0.00	0.00	3.00	0.00	0.00
VAS at rest	52		PRP	27	0.11	0.58	0.00	0.00	3.00	0.00	0.00
PPT	0	N	PRP	30	25.38	11.76	22.21	3.63	59.92	18.14	33.15
PPT	4	N	PRP	30	28.06	11.81	25.20	13.83	70.90	20.69	30.60
PPT	12	N	PRP	30	30.44	14.28	28.39	10.89	72.47	21.67	34.13
PPT	24	N	PRP	29	37.18	12.68	35.50	12.75	71.29	29.22	41.48
PPT	52	N	PRP	27	39.13	13.15	39.23	14.34	68.65	27.46	47.17
Cozen's test	0	VAS	PRP	30	5.42	2.55	5.00	0.00	10.00	5.00	7.50
Cozen's test	4	VAS	PRP	30	3.33	2.21	2.50	0.00	7.50	2.50	5.00
Cozen's test	12	VAS	PRP	30	1.95	2.58	0.50	0.00	10.00	0.00	2.50
Cozen's test	24	VAS	PRP	29	0.52	1.30	0.00	0.00	5.00	0.00	0.00
Cozen's test	52	VAS	PRP	27	0.19	0.96	0.00	0.00	5.00	0.00	0.00
Thomson's test	0	VAS	PRP	30	7.50	2.18	7.50	2.50	10.00	7.50	10.00
Thomson's test	4	VAS	PRP	30	6.00	2.51	7.50	0.00	10.00	5.00	7.50
Thomson's test	12	VAS	PRP	30	3.73	2.50	4.00	0.00	8.00	2.50	5.00
Thomson's test	24	VAS	PRP	29	1.69	2.18	1.00	0.00	7.50	0.00	2.00
Thomson's test	52	VAS	PRP	27	1.26	2.12	0.00	0.00	8.00	0.00	1.00
Mill's test	0	VAS	PRP	30	4.25	3.09	5.00	0.00	10.00	2.50	7.50
Mill's test	4	VAS	PRP	30	2.08	2.37	2.50	0.00	7.50	0.00	2.50

Mill's test	12	VAS	PRP	30	1.20	1.99	0.00	0.00	7.50	0.00	2.50
Mill's test	24	VAS	PRP	29	0.29	0.80	0.00	0.00	3.00	0.00	0.00
Mill's test	52	VAS	PRP	27	0.09	0.48	0.00	0.00	2.50	0.00	0.00
Maudsley's test	0	VAS	PRP	30	6.42	2.24	7.50	2.50	10.00	5.00	7.50
Maudsley's test	4	VAS	PRP	30	4.83	2.62	5.00	0.00	10.00	2.50	7.50
Maudsley's test	12	VAS	PRP	30	3.40	2.45	2.50	0.00	7.50	1.50	5.00
Maudsley's test	24	VAS	PRP	29	1.47	2.04	1.00	0.00	7.50	0.00	2.00
Maudsley's test	52	VAS	PRP	27	1.33	2.15	0.00	0.00	8.00	0.00	3.00
Chair test	0	VAS	PRP	30	6.00	2.24	6.25	0.00	10.00	5.00	7.50
Chair test	4	VAS	PRP	30	4.83	2.62	5.00	0.00	10.00	2.50	7.50
Chair test	12	VAS	PRP	30	2.65	2.63	2.50	0.00	7.50	0.00	5.00
Chair test	24	VAS	PRP	29	1.52	2.16	1.00	0.00	7.50	0.00	2.00
Chair test	52	VAS	PRP	27	0.89	1.71	0.00	0.00	6.00	0.00	1.00
PRTEE	0		PRP	30	45.98	19.77	41.25	19.50	93.50	32.50	52.00
PRTEE	4		PRP	30	28.73	17.37	23.25	3.00	64.50	17.50	39.50
PRTEE	12		PRP	30	18.71	16.30	13.50	0.50	75.00	9.00	21.00
PRTEE	24		PRP	29	10.72	12.35	5.50	0.00	57.50	3.50	17.00
PRTEE	52		PRP	27	5.48	8.53	3.50	0.00	35.50	0.00	5.50
age	0	year	CS	10	47.60	8.81	49.00	32.00	60.00	42.00	55.00
body weight	0	kg	CS	10	86.90	18.00	81.50	65.00	130.00	78.00	93.00
body height	0	m	CS	10	1.73	0.07	1.74	1.64	1.84	1.66	1.79
BMI	0	kg/m^2	CS	10	28.92	5.05	29.03	20.52	38.40	25.47	33.06
duration of symptoms	0	month	CS	10	10.00	11.05	5.00	3.00	36.00	4.00	9.00
VAS	0		CS	10	6.75	1.14	7.00	4.50	9.00	6.00	7.00
VAS	1		CS	10	2.15	2.00	2.00	0.00	7.00	1.00	3.00
VAS	4		CS	10	3.00	1.89	2.50	0.00	6.00	2.00	4.00
VAS	12		CS	9	3.89	2.15	4.00	0.00	8.00	4.00	4.00
VAS	24		CS	9	2.28	2.59	2.00	0.00	8.00	0.00	3.00
VAS	52		CS	9	0.94	1.63	0.00	0.00	5.00	0.00	1.00
VAS at rest	0		CS	10	4.50	1.58	4.50	2.00	7.00	3.00	6.00
VAS at rest	4		CS	10	1.30	1.25	1.50	0.00	3.00	0.00	2.00
VAS at rest	12		CS	9	1.22	1.64	1.00	0.00	5.00	0.00	2.00
VAS at rest	24		CS	9	1.11	2.09	0.00	0.00	6.00	0.00	1.00
VAS at rest	52		CS	9	0.44	1.01	0.00	0.00	3.00	0.00	0.00
PPT	0	N	CS	10	27.97	12.07	25.11	17.65	60.41	21.57	28.44
PPT	4	N	CS	10	32.02	9.84	28.93	23.54	49.92	24.52	33.98
PPT	12	N	CS	9	31.32	12.43	25.50	18.63	52.66	21.57	34.32
PPT	24	N	CS	9	34.20	13.24	31.38	19.52	61.10	24.52	35.99
PPT	52	N	CS	9	36.15	10.16	34.32	25.30	57.00	28.73	38.83
Cozen's test	0	VAS	CS	10	5.75	0.92	5.50	5.00	7.50	5.00	6.00
Cozen's test	4	VAS	CS	10	2.70	1.75	2.75	0.00	5.00	2.00	4.00
Cozen's test	12	VAS	CS	9	3.22	1.64	3.00	1.00	6.00	2.00	4.00
Cozen's test	24	VAS	CS	9	1.61	1.87	1.50	0.00	5.00	0.00	2.00

Cozen's test	52	VAS	CS	9	0.33	0.71	0.00	0.00	2.00	0.00	0.00
Thomson's test	0	VAS	CS	10	6.20	2.10	6.50	2.00	9.00	5.00	8.00
Thomson's test	4	VAS	CS	10	3.15	2.45	2.75	0.00	7.00	2.00	6.00
Thomson's test	12	VAS	CS	9	3.50	1.27	4.00	2.00	6.00	2.50	4.00
Thomson's test	24	VAS	CS	9	2.78	2.49	2.00	0.00	7.00	1.00	4.00
Thomson's test	52	VAS	CS	9	0.89	1.17	0.00	0.00	3.00	0.00	2.00
Mill's test	0	VAS	CS	10	3.55	2.54	4.00	0.00	7.50	1.00	5.00
Mill's test	4	VAS	CS	10	1.60	1.71	1.50	0.00	5.00	0.00	3.00
Mill's test	12	VAS	CS	9	0.44	0.88	0.00	0.00	2.00	0.00	0.00
Mill's test	24	VAS	CS	9	0.11	0.33	0.00	0.00	1.00	0.00	0.00
Mill's test	52	VAS	CS	9	0.22	0.44	0.00	0.00	1.00	0.00	0.00
Maudsley's test	0	VAS	CS	10	6.80	1.40	7.00	4.00	9.00	6.00	8.00
Maudsley's test	4	VAS	CS	10	3.05	2.48	2.25	0.00	6.00	1.00	6.00
Maudsley's test	12	VAS	CS	9	2.89	1.82	2.50	0.00	6.00	2.00	4.00
Maudsley's test	24	VAS	CS	9	2.11	1.90	2.00	0.00	6.00	1.00	2.00
Maudsley's test	52	VAS	CS	9	0.78	0.97	0.00	0.00	2.00	0.00	2.00
Chair test	0	VAS	CS	10	6.00	1.62	6.50	3.00	8.00	5.00	7.00
Chair test	4	VAS	CS	10	3.75	2.20	3.25	0.00	7.00	2.00	6.00
Chair test	12	VAS	CS	9	3.89	1.17	4.00	2.00	6.00	3.00	4.00
Chair test	24	VAS	CS	9	2.39	2.34	2.00	0.00	7.00	0.00	4.00
Chair test	52	VAS	CS	9	0.56	0.88	0.00	0.00	2.00	0.00	1.00
PRTEE	0		CS	10	57.55	16.63	56.00	28.50	87.50	48.00	69.00
PRTEE	4		CS	10	36.70	23.03	36.50	1.00	84.00	20.00	43.00
PRTEE	12		CS	9	31.83	22.67	25.50	13.00	84.00	15.50	32.00
PRTEE	24		CS	9	24.89	24.60	24.50	0.50	81.00	10.00	30.50
PRTEE	52		CS	9	13.94	23.94	2.00	0.00	74.50	0.50	15.00
age	0	year	HA	10	48.60	6.90	48.00	40.00	60.00	42.00	53.00
body weight	0	kg	HA	10	79.50	14.97	78.00	55.00	110.00	70.00	90.00
body height	0	m	HA	10	1.73	0.06	1.73	1.60	1.80	1.69	1.79
BMI	0	kg/m²	HA	10	26.61	4.66	26.04	21.48	36.75	23.46	28.09
duration of symptoms	0	month	HA	10	6.90	6.38	5.00	3.00	24.00	3.00	8.00
VAS	0		HA	10	6.20	1.75	6.00	3.00	8.00	5.00	8.00
VAS	1		HA	10	4.90	2.60	4.50	1.00	9.00	3.00	7.00
VAS	4		HA	10	4.50	1.72	5.00	2.00	8.00	4.00	5.00
VAS	12		HA	10	3.10	1.91	3.00	0.00	7.00	2.00	4.00
VAS	24		HA	8	1.63	1.51	2.00	0.00	4.00	0.00	2.50
VAS	52		HA	8	0.00	0.00	0.00	0.00	0.00	0.00	0.00
VAS at rest	0		HA	10	3.85	1.80	4.00	1.00	6.00	2.00	6.00
VAS at rest	4		HA	10	2.80	2.04	2.00	0.00	7.00	2.00	4.00
VAS at rest	12		HA	10	1.90	2.08	1.50	0.00	6.00	0.00	2.00
VAS at rest	24		HA	8	0.88	1.36	0.50	0.00	4.00	0.00	1.00
VAS at rest	52		HA	8	0.00	0.00	0.00	0.00	0.00	0.00	0.00
PPT	0	N	HA	10	24.57	8.31	23.72	13.14	37.66	18.93	30.99

PPT	4	N	HA	10	24.46	8.86	20.59	15.20	39.13	17.95	34.91
PPT	12	N	HA	10	24.92	5.12	23.64	18.34	34.32	20.59	28.64
PPT	24	N	HA	8	33.23	4.27	33.98	25.20	38.64	31.12	35.89
PPT	52	N	HA	8	33.86	4.91	34.20	25.20	40.89	31.38	36.82
Cozen's test	0	VAS	HA	10	5.65	2.58	6.25	0.00	8.00	5.00	8.00
Cozen's test	4	VAS	HA	10	4.00	2.36	5.00	0.00	7.00	2.00	6.00
Cozen's test	12	VAS	HA	10	2.00	2.58	1.00	0.00	7.00	0.00	3.00
Cozen's test	24	VAS	HA	8	1.00	1.60	0.00	0.00	4.00	0.00	2.00
Cozen's test	52	VAS	HA	8	0.00	0.00	0.00	0.00	0.00	0.00	0.00
Thomson's test	0	VAS	HA	10	7.10	2.33	8.00	1.00	9.00	7.00	8.00
Thomson's test	4	VAS	HA	10	6.15	2.36	7.00	2.00	8.00	5.50	8.00
Thomson's test	12	VAS	HA	10	4.20	2.66	5.00	0.00	7.00	3.00	6.00
Thomson's test	24	VAS	HA	8	2.25	1.98	3.00	0.00	5.00	0.00	3.50
Thomson's test	52	VAS	HA	8	0.50	0.53	0.50	0.00	1.00	0.00	1.00
Mill's test	0	VAS	HA	10	4.10	2.73	4.50	0.00	8.00	2.00	6.00
Mill's test	4	VAS	HA	10	3.30	2.49	3.25	0.00	7.00	1.00	5.00
Mill's test	12	VAS	HA	10	2.25	1.44	2.00	0.00	5.00	1.50	3.00
Mill's test	24	VAS	HA	8	1.00	1.77	0.00	0.00	5.00	0.00	1.50
Mill's test	52	VAS	HA	8	0.00	0.00	0.00	0.00	0.00	0.00	0.00
Maudsley's test	0	VAS	HA	10	7.00	1.83	7.50	4.00	9.00	6.00	8.00
Maudsley's test	4	VAS	HA	10	5.75	2.62	6.50	1.00	8.00	4.00	8.00
Maudsley's test	12	VAS	HA	10	3.85	2.16	3.00	0.50	7.00	3.00	6.00
Maudsley's test	24	VAS	HA	8	2.50	1.77	3.00	0.00	5.00	1.00	3.50
Maudsley's test	52	VAS	HA	8	0.38	0.52	0.00	0.00	1.00	0.00	1.00
Chair test	0	VAS	HA	10	6.80	1.06	7.00	5.00	8.00	6.50	7.50
Chair test	4	VAS	HA	10	5.80	2.20	6.50	2.00	8.00	5.00	7.00
Chair test	12	VAS	HA	10	4.40	2.67	4.50	0.00	8.00	3.00	7.00
Chair test	24	VAS	HA	8	1.75	1.83	1.50	0.00	5.00	0.00	3.00
Chair test	52	VAS	HA	8	0.38	0.52	0.00	0.00	1.00	0.00	1.00
PRTEE	0		HA	10	51.90	17.44	56.00	14.50	75.50	46.50	60.00
PRTEE	4		HA	10	38.15	22.00	36.00	5.00	69.00	24.00	59.00
PRTEE	12		HA	10	28.30	21.80	20.50	1.00	66.00	12.50	53.50
PRTEE	24		HA	8	13.50	14.37	11.00	0.00	41.50	1.50	20.75
PRTEE	52		HA	8	1.75	1.71	1.25	0.00	5.00	0.50	2.75
age	0	year	PL	10	44.90	9.76	48.00	31.00	60.00	36.00	50.00
body weight	0	kg	PL	10	81.70	16.18	85.00	54.00	105.00	65.00	94.00
body height	0	m	PL	10	1.71	0.10	1.72	1.52	1.86	1.65	1.77
BMI	0	kg/m²	PL	10	27.79	4.53	27.38	21.89	35.49	24.15	30.35
duration of symptoms	0	month	PL	10	30.50	73.90	5.00	3.00	240.00	3.00	12.00
VAS	0		PL	10	5.70	1.81	5.75	2.00	8.00	5.00	7.00
VAS	1		PL	10	4.90	2.23	5.00	1.00	8.00	3.00	7.00
VAS	4		PL	10	3.25	2.68	2.75	0.00	9.00	2.00	5.00
VAS	12		PL	10	2.55	2.75	2.25	0.00	8.00	0.00	5.00

VAS	24		PL	10	1.60	2.84	0.00	0.00	9.00	0.00	2.00
VAS	52		PL	10	0.90	2.23	0.00	0.00	7.00	0.00	0.00
VAS at rest	0		PL	10	3.00	2.98	3.00	0.00	8.00	0.00	5.00
VAS at rest	4		PL	10	1.90	2.73	0.50	0.00	8.00	0.00	3.00
VAS at rest	12		PL	10	1.70	2.63	0.00	0.00	7.00	0.00	4.00
VAS at rest	24		PL	10	1.00	1.94	0.00	0.00	6.00	0.00	2.00
VAS at rest	52		PL	10	0.50	1.08	0.00	0.00	3.00	0.00	0.00
PPT	0	N	PL	10	28.82	16.49	25.11	8.73	67.96	19.81	37.27
PPT	4	N	PL	10	28.80	16.86	27.12	9.61	72.57	19.52	30.89
PPT	12	N	PL	10	32.05	13.52	27.95	16.38	55.70	22.16	37.27
PPT	24	N	PL	10	37.61	13.62	33.39	19.81	60.80	28.24	48.05
PPT	52	N	PL	10	42.51	11.91	37.66	29.42	59.82	31.97	56.00
Cozen's test	0	VAS	PL	10	4.85	2.38	5.00	0.00	9.00	5.00	5.00
Cozen's test	4	VAS	PL	10	2.85	1.86	3.00	0.00	5.00	2.00	5.00
Cozen's test	12	VAS	PL	10	0.90	1.52	0.00	0.00	4.00	0.00	2.00
Cozen's test	24	VAS	PL	10	0.45	0.96	0.00	0.00	2.50	0.00	0.00
Cozen's test	52	VAS	PL	10	0.00	0.00	0.00	0.00	0.00	0.00	0.00
Thomson's test	0	VAS	PL	10	6.50	1.93	7.25	2.00	9.00	6.00	7.50
Thomson's test	4	VAS	PL	10	4.25	2.83	4.00	0.00	8.00	2.50	7.00
Thomson's test	12	VAS	PL	10	3.75	2.32	3.00	0.00	7.00	2.00	6.00
Thomson's test	24	VAS	PL	10	1.50	2.01	0.50	0.00	6.00	0.00	3.00
Thomson's test	52	VAS	PL	10	0.20	0.63	0.00	0.00	2.00	0.00	0.00
Mill's test	0	VAS	PL	10	5.80	1.95	5.00	2.50	8.00	5.00	8.00
Mill's test	4	VAS	PL	10	0.90	1.26	0.00	0.00	3.00	0.00	2.50
Mill's test	12	VAS	PL	10	1.30	2.58	0.00	0.00	8.00	0.00	2.00
Mill's test	24	VAS	PL	10	1.00	2.31	0.00	0.00	7.00	0.00	0.00
Mill's test	52	VAS	PL	10	0.00	0.00	0.00	0.00	0.00	0.00	0.00
Maudsley's test	0	VAS	PL	10	5.90	2.47	6.50	1.00	9.00	5.00	7.50
Maudsley's test	4	VAS	PL	10	3.70	2.74	3.00	0.00	8.00	2.50	6.00
Maudsley's test	12	VAS	PL	10	3.65	2.65	2.75	0.00	8.00	2.00	6.00
Maudsley's test	24	VAS	PL	10	1.60	2.32	0.00	0.00	6.00	0.00	3.00
Maudsley's test	52	VAS	PL	10	0.40	1.26	0.00	0.00	4.00	0.00	0.00
Chair test	0	VAS	PL	10	5.85	1.67	6.50	2.00	7.50	5.00	7.00
Chair test	4	VAS	PL	10	4.30	2.63	5.00	0.00	7.00	2.00	7.00
Chair test	12	VAS	PL	10	3.25	2.55	2.75	0.00	6.00	1.00	6.00
Chair test	24	VAS	PL	10	1.20	1.99	0.00	0.00	6.00	0.00	2.00
Chair test	52	VAS	PL	10	0.50	1.58	0.00	0.00	5.00	0.00	0.00
PRTEE	0		PL	10	45.70	18.25	39.25	22.50	84.00	35.50	56.00
PRTEE	4		PL	10	30.75	24.53	26.50	6.00	84.00	11.00	48.50
PRTEE	12		PL	10	24.10	23.93	13.50	4.50	81.00	8.00	39.00
PRTEE	24		PL	10	14.75	24.32	2.25	0.00	74.50	0.00	26.50
PRTEE	52		PL	10	8.70	19.97	0.00	0.00	61.00	0.00	1.00

All, all groups combined; BMI, Body Mass Index; CS, corticosteroid injection group; HA, hyaluronic acid injection group; PL, placebo injection group; PPT, Pressure Pain Threshold; PRP, platelet-rich plasma injection group; PRTEE, Patient-rated Tennis Elbow Evaluation; VAS, Visual Analogue Scale.

Supplementary materials – Table S2: secondary outcomes

	Follow-up (weeks)	unit	Group	*n*	Mean	SD	Median	Minimum	Maximum	Q1	Q2
SEV	0	%	All	60	47.08	15.38	50.00	0.00	80.00	40.00	50.00
SEV	4	%	All	60	65.08	17.72	70.00	20.00	95.00	50.00	80.00
SEV	12	%	All	59	69.97	20.63	75.00	10.00	100.00	50.00	90.00
SEV	24	%	All	56	82.46	18.23	90.00	20.00	100.00	80.00	95.00
SEV	52	%	All	54	88.78	14.93	90.00	30.00	100.00	90.00	99.00
grip strength	0	MAX kg	All	60	33.34	14.03	30.00	8.00	62.50	22.00	46.00
grip strength	4	MAX kg	All	60	34.99	13.73	34.00	12.00	60.00	22.00	47.00
grip strength	12	MAX kg	All	59	37.59	13.87	37.50	8.00	67.90	28.00	50.00
grip strength	24	MAX kg	All	56	41.06	13.88	39.50	20.00	69.00	28.75	52.00
grip strength	52	MAX kg	All	54	42.11	14.47	39.50	18.24	81.00	30.00	55.00
key-pinch strength	0	MAX kg	All	60	9.16	3.09	9.40	4.00	16.00	6.40	11.50
key-pinch strength	4	MAX kg	All	60	9.50	2.84	9.60	4.50	16.00	7.55	11.13
key-pinch strength	12	MAX kg	All	59	9.50	2.67	9.30	4.75	16.00	7.50	11.50
key-pinch strength	24	MAX kg	All	56	9.73	2.70	9.45	4.00	16.50	7.60	12.00
key-pinch strength	52	MAX kg	All	54	10.00	2.33	9.85	5.50	16.50	8.00	12.00
strength of elbow flexion	0	N	All	60	242.95	89.74	229.48	100.03	435.42	170.15	305.48
strength of elbow flexion	4	N	All	60	237.90	85.70	209.86	84.34	402.07	166.71	309.89
strength of elbow flexion	12	N	All	59	246.39	85.28	229.97	104.93	425.61	174.56	317.74
strength of elbow flexion	24	N	All	56	260.38	88.81	241.49	134.35	442.28	195.15	319.21
strength of elbow flexion	52	N	All	54	268.43	96.18	241.66	138.27	542.31	187.31	354.02
strength of elbow extension	0	N	All	60	176.00	59.44	167.69	76.49	343.23	129.94	208.39
strength of elbow extension	4	N	All	60	172.90	49.71	167.53	76.49	326.56	129.45	205.45
strength of elbow extension	12	N	All	59	177.43	54.01	165.73	68.65	294.20	133.37	217.71
strength of elbow extension	24	N	All	56	182.40	57.08	174.56	86.30	355.98	138.76	217.71
strength of elbow extension	52	N	All	54	181.99	55.28	168.27	81.40	354.02	137.29	225.55
strength of wrist extension	0	N	All	60	114.05	49.92	107.38	25.50	238.30	84.34	137.78
strength of wrist extension	4	N	All	60	126.82	45.44	127.49	32.36	224.57	97.09	151.02

strength of wrist extension	12	N	All	59	140.71	47.19	141.22	27.46	229.48	107.87	167.69
strength of wrist extension	24	N	All	56	157.32	47.26	149.06	62.76	253.01	123.07	193.19
strength of wrist extension	52	N	All	54	169.08	48.64	160.99	63.74	291.26	137.29	209.86
strength of wrist flexion	0	N	All	60	158.01	50.26	150.94	66.69	368.73	120.62	191.23
strength of wrist flexion	4	N	All	60	159.41	43.64	159.85	80.41	256.93	127.00	194.66
strength of wrist flexion	12	N	All	59	165.57	51.15	158.18	72.57	265.76	122.58	203.00
strength of wrist flexion	24	N	All	56	173.66	49.43	166.71	74.53	295.18	137.29	201.01
strength of wrist flexion	52	N	All	54	175.71	49.34	170.15	81.30	276.55	134.35	215.75
strength of forearm supination	0	N	All	60	24.48	10.75	23.45	0.98	61.78	17.16	30.40
strength of forearm supination	4	N	All	60	25.40	10.64	23.54	0.98	59.82	18.31	33.34
strength of forearm supination	12	N	All	59	28.63	13.25	24.52	4.90	77.47	19.61	34.32
strength of forearm supination	24	N	All	56	31.51	11.43	30.20	10.00	59.82	23.54	38.25
strength of forearm supination	52	N	All	54	32.18	11.86	32.85	13.73	62.76	23.54	39.23
strength of forearm pronation	0	N	All	60	41.36	18.71	38.74	13.73	90.22	25.50	53.94
strength of forearm pronation	4	N	All	60	43.70	17.83	41.68	11.77	84.34	28.44	57.86
strength of forearm pronation	12	N	All	59	46.40	17.18	44.13	15.69	84.34	31.38	58.84
strength of forearm pronation	2	N	All	56	50.02	20.40	44.13	19.61	98.07	31.87	66.69
strength of forearm pronation	52	N	All	54	51.37	18.14	49.52	21.20	96.11	38.25	63.74
DASH	0		All	60	40.25	17.23	35.83	13.33	79.17	27.50	52.07
DASH	4		All	60	27.83	18.10	23.33	0.00	75.00	13.75	37.50
DASH	12		All	59	19.79	17.05	13.33	0.00	75.00	7.50	28.33
DASH	24		All	56	11.73	15.83	6.67	0.00	74.17	1.67	15.42
DASH	52		All	54	6.16	12.25	1.67	0.00	71.67	0.00	5.83
SEV	0	%	PRP	30	48.50	16.56	50.00	0.00	80.00	40.00	50.00
SEV	4	%	PRP	30	66.17	14.95	70.00	30.00	90.00	50.00	75.00
SEV	12	%	PRP	30	72.77	21.44	80.00	10.00	98.00	50.00	90.00
SEV	24	%	PRP	29	86.24	14.99	90.00	30.00	100.00	80.00	95.00

SEV	52	%	PRP	27	88.52	15.84	90.00	30.00	100.00	90.00	98.00
grip strength	0	MAX kg	PRP	30	32.45	14.28	30.00	8.00	60.00	22.00	44.00
grip strength	4	MAX kg	PRP	30	33.43	13.63	30.00	12.00	55.00	22.00	46.00
grip strength	12	MAX kg	PRP	30	35.63	14.49	35.00	8.00	60.00	22.00	50.00
grip strength	24	MAX kg	PRP	29	39.45	14.22	36.00	20.00	63.00	28.50	50.00
grip strength	52	MAX kg	PRP	27	41.16	16.78	38.50	18.24	81.00	28.60	59.00
key-pinch strength	0	MAX kg	PRP	30	9.09	3.42	8.88	4.00	16.00	6.00	11.75
key-pinch strength	4	MAX kg	PRP	30	9.68	3.01	9.50	4.50	16.00	7.50	11.25
key-pinch strength	12	MAX kg	PRP	30	9.30	2.96	9.00	4.75	16.00	7.00	11.50
key-pinch strength	24	MAX kg	PRP	29	9.66	3.04	9.00	4.00	16.50	7.25	11.00
key-pinch strength	52	MAX kg	PRP	27	9.96	2.71	10.00	5.50	16.50	8.00	12.00
strength of elbow flexion	0	N	PRP	30	234.97	90.34	220.16	101.01	420.71	147.10	302.04
strength of elbow flexion	4	N	PRP	30	236.83	91.14	208.39	84.34	398.15	165.73	313.81
strength of elbow flexion	12	N	PRP	30	246.44	84.57	217.71	104.93	425.61	174.56	318.72
strength of elbow flexion	24	N	PRP	29	254.63	93.82	227.51	134.35	442.28	172.60	314.79
strength of elbow flexion	52	N	PRP	27	258.19	92.92	227.51	139.21	420.71	176.34	343.23
strength of elbow extension	0	N	PRP	30	175.96	66.71	160.34	76.49	343.23	128.47	215.75
strength of elbow extension	4	N	PRP	30	172.07	54.51	155.44	76.49	326.56	129.45	207.90
strength of elbow extension	12	N	PRP	30	172.17	56.83	159.36	68.65	294.20	132.39	217.71
strength of elbow extension	24	N	PRP	29	176.89	57.05	168.67	86.30	322.64	139.25	209.86
strength of elbow extension	52	N	PRP	27	177.08	57.04	160.83	81.40	282.43	129.45	229.48
strength of wrist extension	0	N	PRP	30	116.44	52.47	109.34	25.50	227.51	78.45	158.87
strength of wrist extension	4	N	PRP	30	121.50	51.14	107.87	47.07	224.57	90.22	151.02
strength of wrist extension	12	N	PRP	30	140.46	49.45	151.02	27.46	225.55	101.01	177.50
strength of wrist extension	24	N	PRP	29	159.56	51.16	152.00	76.49	253.01	121.60	200.00
strength of wrist extension	52	N	PRP	27	172.99	53.26	160.83	72.81	291.26	138.27	215.75
strength of wrist flexion	0	N	PRP	30	159.03	61.28	146.61	66.69	368.73	118.66	199.07
strength of wrist flexion	4	N	PRP	30	151.81	48.27	141.71	80.41	246.15	116.70	193.19

strength of wrist flexion	12	N	PRP	30	162.07	59.05	153.96	72.57	265.76	114.74	200.06
strength of wrist flexion	24	N	PRP	29	174.03	60.40	164.75	74.53	295.18	127.49	200.00
strength of wrist flexion	52	N	PRP	27	175.26	60.08	167.69	81.30	276.55	126.51	221.63
strength of forearm supination	0	N	PRP	30	26.05	12.13	23.54	6.86	61.78	16.67	32.36
strength of forearm supination	4	N	PRP	30	24.71	10.54	23.54	0.98	47.07	17.65	33.34
strength of forearm supination	12	N	PRP	30	29.05	15.74	23.05	4.90	77.47	18.63	33.15
strength of forearm supination	24	N	PRP	29	32.05	14.39	28.44	10.00	59.82	21.57	39.23
strength of forearm supination	52	N	PRP	27	31.19	15.11	24.52	13.73	62.76	20.59	42.17
strength of forearm pronation	0	N	PRP	30	36.64	18.35	27.95	13.73	90.22	24.52	50.01
strength of forearm pronation	4	N	PRP	30	40.24	17.93	33.34	13.73	76.49	26.48	55.90
strength of forearm pronation	12	N	PRP	30	46.44	20.15	40.70	15.69	84.34	28.44	60.80
strength of forearm pronation	24	N	PRP	29	48.44	22.56	42.00	19.61	98.07	30.00	67.67
strength of forearm pronation	52	N	PRP	27	49.46	19.84	46.09	21.20	93.16	32.36	63.74
DASH	0		PRP	30	37.28	17.17	33.75	13.33	79.17	22.50	47.50
DASH	4		PRP	30	24.78	16.04	18.75	0.83	65.83	12.50	36.67
DASH	12		PRP	30	15.17	12.32	12.08	0.83	53.33	7.50	17.50
DASH	24		PRP	29	7.90	9.10	5.83	0.00	40.83	1.67	10.83
DASH	52		PRP	27	4.75	6.95	3.33	0.00	28.33	0.00	5.83
SEV	0	%	CS	10	45.00	12.69	45.00	30.00	60.00	30.00	60.00
SEV	4	%	CS	10	71.00	15.24	70.00	40.00	90.00	60.00	80.00
SEV	12	%	CS	9	64.44	16.85	65.00	40.00	95.00	50.00	70.00
SEV	24	%	CS	9	71.67	20.92	70.00	30.00	95.00	70.00	90.00
SEV	52	%	CS	9	82.22	18.05	90.00	40.00	100.00	80.00	90.00
grip strength	0	MAX kg	CS	10	34.66	15.23	34.50	18.00	62.50	20.30	48.00
grip strength	4	MAX kg	CS	10	37.51	12.35	36.00	22.00	60.00	28.50	49.00
grip strength	12	MAX kg	CS	9	37.50	12.21	37.50	23.00	54.00	28.00	50.00
grip strength	24	MAX kg	CS	9	38.63	12.51	39.00	24.00	56.00	28.00	50.00
grip strength	52	MAX kg	CS	9	38.58	11.69	38.00	25.00	56.00	28.00	51.00

key-pinch strength	0	MAX kg	CS	10	9.48	3.14	9.80	5.00	15.00	7.50	11.50
key-pinch strength	4	MAX kg	CS	10	9.69	2.68	10.05	5.50	13.50	7.60	11.75
key-pinch strength	12	MAX kg	CS	9	9.51	2.42	9.60	6.20	14.00	7.80	11.00
key-pinch strength	24	MAX kg	CS	9	9.42	2.56	9.40	6.00	12.50	7.70	12.00
key-pinch strength	52	MAX kg	CS	9	9.70	2.29	9.70	6.20	13.00	8.00	10.50
strength of elbow flexion	0	N	CS	10	237.55	71.30	220.32	162.79	358.92	176.52	286.35
strength of elbow flexion	4	N	CS	10	220.16	62.00	202.02	147.10	340.29	180.44	271.64
strength of elbow flexion	12	N	CS	9	232.25	73.57	229.97	114.74	343.23	176.52	288.32
strength of elbow flexion	24	N	CS	9	242.17	75.17	241.73	149.06	381.48	181.42	294.20
strength of elbow flexion	52	N	CS	9	243.53	88.01	244.04	138.27	369.71	171.62	299.10
strength of elbow extension	0	N	CS	10	171.94	28.81	176.68	125.53	205.94	146.12	197.11
strength of elbow extension	4	N	CS	10	175.70	34.55	173.41	117.68	224.57	150.04	202.02
strength of elbow extension	12	N	CS	9	178.75	39.13	176.03	110.82	245.17	163.77	198.09
strength of elbow extension	24	N	CS	9	186.00	47.01	181.42	117.68	267.72	154.95	219.67
strength of elbow extension	52	N	CS	9	173.14	35.13	165.73	125.53	235.36	159.85	196.13
strength of wrist extension	0	N	CS	10	110.36	19.54	108.04	72.57	137.29	101.99	128.47
strength of wrist extension	4	N	CS	10	136.90	25.59	131.41	88.26	171.62	127.49	163.77
strength of wrist extension	12	N	CS	9	122.47	29.51	129.45	57.86	166.71	120.62	133.37
strength of wrist extension	24	N	CS	9	133.28	28.64	135.50	98.07	177.50	114.74	141.22
strength of wrist extension	52	N	CS	9	147.89	30.18	137.29	112.78	196.13	123.56	170.00
strength of wrist flexion	0	N	CS	10	143.26	19.99	135.74	117.68	176.52	128.47	164.75
strength of wrist flexion	4	N	CS	10	168.84	39.88	168.02	128.47	256.93	132.39	195.15
strength of wrist flexion	12	N	CS	9	152.06	21.10	146.61	122.58	197.11	141.22	162.79
strength of wrist flexion	24	N	CS	9	159.10	27.45	146.12	137.29	206.92	137.29	166.71
strength of wrist flexion	52	N	CS	9	159.54	36.43	147.10	115.72	217.71	134.35	196.13
strength of forearm supination	0	N	CS	10	25.48	6.96	25.50	14.71	34.32	22.56	30.40
strength of forearm supination	4	N	CS	10	31.33	10.87	29.42	21.57	59.82	23.54	33.34

strength of forearm supination	12	N	CS	9	26.68	7.30	26.31	16.67	42.17	21.57	29.42
strength of forearm supination	24	N	CS	9	31.62	5.94	31.38	22.56	44.13	30.40	32.36
strength of forearm supination	52	N	CS	9	33.48	4.19	33.95	24.52	39.23	33.34	35.00
strength of forearm pronation	0	N	CS	10	47.69	16.26	46.58	24.52	75.51	36.28	50.99
strength of forearm pronation	4	N	CS	10	49.98	16.64	46.09	34.32	84.34	37.27	56.88
strength of forearm pronation	12	N	CS	9	46.64	12.41	47.07	31.38	71.59	39.23	50.99
strength of forearm pronation	24	N	CS	9	51.32	15.45	44.13	34.32	76.49	39.23	59.82
strength of forearm pronation	52	N	CS	9	50.21	14.62	50.01	27.46	71.59	40.21	58.84
DASH	0		CS	10	49.68	15.09	48.58	28.33	78.00	41.67	58.30
DASH	4		CS	10	32.50	19.56	30.42	0.00	75.00	23.33	35.83
DASH	12		CS	9	28.98	19.50	25.83	12.50	75.00	16.67	29.17
DASH	24		CS	9	21.11	22.38	17.50	2.50	74.17	6.67	23.33
DASH	52		CS	9	13.61	23.26	1.67	0.00	71.67	0.00	16.67
SEV	0	%	HA	10	46.00	14.87	47.50	25.00	80.00	40.00	50.00
SEV	4	%	HA	10	57.00	19.47	50.00	30.00	90.00	50.00	60.00
SEV	12	%	HA	10	69.50	15.89	67.50	50.00	100.00	60.00	80.00
SEV	24	%	HA	8	83.38	11.64	82.50	60.00	97.00	80.00	92.50
SEV	52	%	HA	8	95.50	4.04	95.00	90.00	100.00	92.50	99.50
grip strength	0	MAX kg	HA	10	31.81	9.28	31.60	18.00	48.90	23.20	38.00
grip strength	4	MAX kg	HA	10	31.70	14.22	31.05	13.50	56.00	17.90	40.00
grip strength	12	MAX kg	HA	10	35.68	10.09	35.20	18.00	50.40	28.00	43.50
grip strength	24	MAX kg	HA	8	40.30	10.21	41.95	20.50	52.00	35.00	48.00
grip strength	52	MAX kg	HA	8	40.68	10.55	40.50	24.10	58.00	34.00	47.15
key-pinch strength	0	MAX kg	HA	10	8.29	1.35	8.50	6.30	10.00	7.00	9.50
key-pinch strength	4	MAX kg	HA	10	8.26	2.32	8.90	4.70	11.00	5.90	10.00
key-pinch strength	12	MAX kg	HA	10	9.09	1.50	9.40	6.00	11.10	8.20	10.00
key-pinch strength	24	MAX kg	HA	8	9.29	1.29	9.10	7.20	11.00	8.45	10.50
key-pinch strength	52	MAX kg	HA	8	9.39	0.89	9.30	8.10	11.10	8.95	9.70
strength of elbow flexion	0	N	HA	10	236.44	69.63	223.10	140.24	362.85	186.33	277.53

strength of elbow flexion	4	N	HA	10	219.59	78.65	197.69	124.54	339.31	156.91	294.20
strength of elbow flexion	12	N	HA	10	219.36	73.27	205.86	126.51	328.52	156.91	296.16
strength of elbow flexion	24	N	HA	8	255.22	65.97	232.91	167.69	353.04	209.86	317.74
strength of elbow flexion	52	N	HA	8	279.39	82.99	247.21	196.13	413.84	211.82	353.53
strength of elbow extension	0	N	HA	10	154.23	40.87	159.19	95.12	220.65	122.58	171.62
strength of elbow extension	4	N	HA	10	150.25	35.53	133.45	112.78	205.94	123.56	176.52
strength of elbow extension	12	N	HA	10	162.25	44.46	145.79	121.60	241.24	123.56	201.04
strength of elbow extension	24	N	HA	8	163.26	34.43	153.88	129.45	215.75	136.31	190.25
strength of elbow extension	52	N	HA	8	173.35	33.79	164.83	132.39	225.55	146.12	203.49
strength of wrist extension	0	N	HA	10	97.66	34.63	101.91	43.15	150.04	84.34	114.74
strength of wrist extension	4	N	HA	10	113.90	52.74	123.32	32.36	192.21	68.65	145.14
strength of wrist extension	12	N	HA	10	133.37	49.39	137.29	63.74	215.75	76.49	167.69
strength of wrist extension	24	N	HA	8	158.05	47.27	157.56	62.76	226.53	145.63	184.37
strength of wrist extension	52	N	HA	8	160.62	44.65	165.90	63.74	223.59	156.91	176.03
strength of wrist flexion	0	N	HA	10	153.56	34.69	153.39	102.97	208.88	128.47	179.46
strength of wrist flexion	4	N	HA	10	160.29	33.87	164.51	91.20	196.13	140.24	191.23
strength of wrist flexion	12	N	HA	10	164.59	39.43	160.50	107.87	223.59	137.29	196.13
strength of wrist flexion	24	N	HA	8	174.93	23.96	171.13	142.20	214.77	157.40	192.70
strength of wrist flexion	52	N	HA	8	172.44	17.50	172.79	143.18	196.13	160.34	186.96
strength of forearm supination	0	N	HA	10	20.81	7.11	20.19	7.85	31.38	17.65	26.48
strength of forearm supination	4	N	HA	10	20.23	11.08	20.10	0.98	35.30	17.65	26.48
strength of forearm supination	12	N	HA	10	24.60	4.92	24.52	16.67	34.32	23.54	25.50
strength of forearm supination	24	N	HA	8	26.15	5.85	25.99	14.71	35.30	24.68	28.93
strength of forearm supination	52	N	HA	8	28.48	7.15	26.78	19.61	42.86	24.03	31.87
strength of forearm pronation	0	N	HA	10	41.81	15.39	41.35	16.67	67.67	29.42	54.92

strength of forearm pronation	4	N	HA	10	41.29	16.82	39.23	11.77	65.70	28.44	58.84
strength of forearm pronation	12	N	HA	10	43.75	13.06	42.25	24.52	63.74	34.32	56.88
strength of forearm pronation	24	N	HA	8	45.23	12.73	43.15	28.44	64.72	35.30	55.90
strength of forearm pronation	52	N	HA	8	49.53	11.65	50.01	29.42	66.90	43.58	56.39
DASH	0		HA	10	43.33	17.68	45.83	13.33	67.50	34.17	53.33
DASH	4		HA	10	33.92	20.15	32.08	6.67	65.83	16.67	53.33
DASH	12		HA	10	25.00	20.59	20.00	0.00	61.67	10.00	47.50
DASH	24		HA	8	13.96	14.97	10.00	0.83	40.83	1.67	23.33
DASH	52		HA	8	2.40	2.42	1.67	0.00	7.50	0.83	3.33
SEV	0	%	PL	10	46.00	16.47	50.00	20.00	70.00	40.00	60.00
SEV	4	%	PL	10	64.00	24.70	67.50	20.00	95.00	50.00	80.00
SEV	12	%	PL	10	67.00	26.27	75.00	20.00	90.00	50.00	90.00
SEV	24	%	PL	10	80.50	25.87	90.00	20.00	100.00	65.00	100.00
SEV	52	%	PL	10	90.00	14.14	95.00	60.00	100.00	90.00	100.00
grip strength	0	MAX kg	PL	10	36.20	17.27	44.00	10.00	56.00	20.00	50.00
grip strength	4	MAX kg	PL	10	40.40	14.93	46.00	14.00	56.00	34.00	50.00
grip strength	12	MAX kg	PL	10	45.49	15.58	50.50	16.00	67.90	32.00	55.00
grip strength	24	MAX kg	PL	10	48.50	15.86	52.50	21.00	69.00	34.00	56.00
grip strength	52	MAX kg	PL	10	49.00	11.90	52.50	33.00	60.00	34.00	60.00
key-pinch strength	0	MAX kg	PL	10	9.90	3.42	11.50	4.00	13.50	7.75	12.00
key-pinch strength	4	MAX kg	PL	10	10.00	2.99	10.75	4.50	14.50	8.20	11.75
key-pinch strength	12	MAX kg	PL	10	10.51	2.95	11.50	5.00	13.50	8.00	13.00
key-pinch strength	24	MAX kg	PL	10	10.55	2.77	12.00	5.50	13.50	8.50	12.00
key-pinch strength	52	MAX kg	PL	10	10.88	2.05	11.75	7.50	13.00	8.50	12.50
strength of elbow flexion	0	N	PL	10	278.80	121.83	302.54	100.03	435.42	180.44	393.25
strength of elbow flexion	4	N	PL	10	277.14	94.44	277.53	158.87	402.07	190.25	388.34
strength of elbow flexion	12	N	PL	10	285.96	104.38	276.06	139.25	405.01	186.33	392.27
strength of elbow flexion	24	N	PL	10	297.53	102.03	286.35	156.91	418.74	207.90	402.07
strength of elbow flexion	52	N	PL	10	309.74	119.79	303.03	142.20	542.31	224.14	392.27
strength of elbow extension	0	N	PL	10	201.92	70.91	209.86	104.93	302.04	119.64	240.26

strength of elbow extension	4	N	PL	10	195.25	55.11	191.23	127.49	296.16	157.89	233.40
strength of elbow extension	12	N	PL	10	207.21	60.96	200.06	120.62	291.26	156.91	264.78
strength of elbow extension	24	N	PL	10	210.45	74.74	191.72	112.78	355.98	162.79	248.11
strength of elbow extension	52	N	PL	10	210.11	74.28	196.62	123.56	354.02	137.29	254.97
strength of wrist extension	0	N	PL	10	127.00	73.24	90.71	50.01	238.30	70.61	195.15
strength of wrist extension	4	N	PL	10	145.63	29.76	140.73	103.95	201.04	127.49	167.69
strength of wrist extension	12	N	PL	10	165.22	46.88	151.51	98.07	229.48	142.20	209.86
strength of wrist extension	24	N	PL	10	171.87	47.00	156.71	114.74	249.09	137.29	209.86
strength of wrist extension	52	N	PL	10	184.37	50.58	189.93	112.78	240.00	140.24	235.36
strength of wrist flexion	0	N	PL	10	174.17	47.92	172.60	114.74	250.07	120.62	217.71
strength of wrist flexion	4	N	PL	10	171.91	42.12	176.52	97.09	225.55	152.00	208.88
strength of wrist flexion	12	N	PL	10	189.20	53.24	204.47	98.07	255.95	158.18	216.73
strength of wrist flexion	24	N	PL	10	184.66	46.00	196.13	105.91	229.48	166.71	221.63
strength of wrist flexion	52	N	PL	10	194.11	42.89	212.80	119.64	236.34	167.69	221.00
strength of forearm supination	0	N	PL	10	22.46	12.45	20.59	0.98	45.11	15.69	29.42
strength of forearm supination	4	N	PL	10	26.67	8.42	26.97	14.71	37.27	20.59	34.32
strength of forearm supination	12	N	PL	10	33.17	14.66	36.28	15.89	61.78	19.61	42.17
strength of forearm supination	24	N	PL	10	34.13	7.84	36.77	20.59	43.15	27.46	40.21
strength of forearm supination	52	N	PL	10	36.64	8.62	36.77	22.56	53.94	33.34	40.00
strength of forearm pronation	0	N	PL	10	48.74	22.98	52.96	13.73	79.43	25.50	66.69
strength of forearm pronation	4	N	PL	10	50.21	18.74	56.39	23.54	74.53	28.44	64.72
strength of forearm pronation	12	N	PL	10	48.68	16.52	51.68	24.52	72.57	32.36	61.78
strength of forearm pronation	24	N	PL	10	57.27	23.06	63.25	26.48	93.16	32.36	71.59
strength of forearm pronation	52	N	PL	10	59.03	20.61	60.31	29.42	96.11	41.19	70.00

DASH	0		PL	10	36.67	17.41	32.92	15.83	75.00	26.67	35.83
DASH	4		PL	10	26.25	20.76	20.42	5.83	75.00	14.17	28.33
DASH	12		PL	10	20.17	20.92	14.17	4.17	74.17	5.83	28.33
DASH	24		PL	10	12.58	22.68	1.25	0.00	71.67	0.00	20.00
DASH	52		PL	10	6.25	13.59	0.00	0.00	38.33	0.00	0.00

All, all groups combined; CS, corticosteroid injection group; DASH, Disability of Arm, Shoulder, and Hand Questionnaire; HA, hyaluronic acid injection group; PL, placebo injection group; PRP, platelet-rich plasma injection group; SEV, Subjected Elbow Value.

Supplementary materials - Figure S1: the exercise instruction card for patients

Time Post-Injection	Recommendation	Example Exercises	Exercise Details	Recommended Duration and Repetitions	Remarks
0–1 week	No exercises	–	–	–	Avoidance of overloading activities
> 1 week	Daily stretching exercises for the forearm extensors.	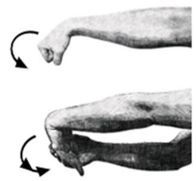	1. The elbow is extended, and the forearm is positioned in pronation (palm facing downward).<br>2. Flex the wrist to its maximum position and hold it for 30 seconds.<br>3. Passively increase wrist flexion (keeping fingers straight) with the assistance of the other hand and hold for an additional 30 seconds.	10 rounds, each lasting 1 minute, with a 30-second interval between rounds.	
> 2 weeks	Eccentric exercises to strengthen the forearm extensors (maintain a daily stretching routine).	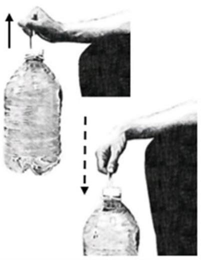	1. While seated on a chair, position the forearm in pronation, resting it stably on the table, with the wrist hanging freely over the table's edge.<br>2. Hold the handle of a bucket or a 5-liter water bottle using an overhand grip.<br>3. With the assistance of the other hand, perform wrist dorsiflexion.<br>4. Engage the muscles actively and slowly lower the bucket by flexing the wrist.	10 rounds of 15 repetitions each, with a 1-minute interval between rounds.	Perform only if the pain does not exceed 3 on the VAS scale during the activity.<br>Begin with a bucket filled with 500 ml of water, gradually increasing the weight every few days.
> 2 months	Return to sport	–	Gradually increase your sporting activity	–	Perform only if no pain occurs during the activity.
